# Molecular evolution of intestinal-type early gastric cancer according to Correa cascade

**DOI:** 10.7555/JBR.38.20240118

**Published:** 2024-09-24

**Authors:** Fangyuan Li, Yaohui Wang, Xiaochun Ping, Jiani C. Yin, Fufeng Wang, Xian Zhang, Xiang Li, Jing Zhai, Lizong Shen

**Affiliations:** 1 Digestive Endoscopy Center, Jiangsu Province Hospital of Chinese Medicine, Affiliated Hospital of Nanjing University of Chinese Medicine, Nanjing, Jiangsu 210029, China; 2 Department of Pathology, Jiangsu Province Hospital of Chinese Medicine, Affiliated Hospital of Nanjing University of Chinese Medicine, Nanjing, Jiangsu 210029, China; 3 Department of General Surgery, the First Affiliated Hospital, Nanjing Medical University, Nanjing, Jiangsu 210029, China; 4 Jiangsu Key Lab of Cancer Biomarkers, Prevention and Treatment, Collaborative Innovation Center for Cancer Personalized Medicine, Nanjing Medical University, Nanjing, Jiangsu 211166, China; 5 Geneseeq Research Institute, Nanjing Geneseeq Technology Inc., Nanjing, Jiangsu 210061, China; 6 Department of Surgical Oncology, Jiangsu Province Hospital of Chinese Medicine, Affiliated Hospital of Nanjing University of Chinese Medicine, Nanjing, Jiangsu 210029, China

**Keywords:** intestinal-type gastric cancer, Correa cascade, gastric intestinal metaplasia, low-grade intraepithelial neoplasia, molecular evolution

## Abstract

Early screening is crucial for the prevention of intestinal-type gastric cancer. The current study aimed to ascertain the molecular evolution of intestinal-type gastric cancer based on the Correa cascade for precise gastric cancer screening. We collected sequential lesions of the Correa cascade in the formalin-fixed and paraffin-embedded endoscopic submucosal dissection (ESD)-resected specimens from 14 Chinese patients by microdissection, and subsequently determined the profiles of somatic aberrations during gastric carcinogenesis using whole-exome sequencing, identifying multiple variants at different Correa stages. The results showed that *TP53*, *PCLO*, and *PRKDC* were the most frequently mutated genes in early gastric cancer (EGC). We found a high frequency of *TP53* alterations in low-grade intraepithelial neoplasia (LGIN), which further increased in high-grade intraepithelial neoplasia (HGIN) and EGC. Intestinal metaplasia (IM) showed no significant correlation with EGC in terms of mutational spectra, whereas both LGIN and HGIN showed higher genomic similarities to EGC, compared with IM. Based on Jaccard similarity coefficients, we constructed three evolutionary models, with most patients showing linear progression from LGIN to HGIN, ultimately resulting in EGC. The extracellular matrix-receptor interaction pathway was revealed to be involved in the linear evolution. Additionally, the retrospective validation study of 39 patients diagnosed with LGIN indicated that *PRKDC* mutations, in addition to *TP53* mutations, may drive LGIN progression to HGIN or EGC. In conclusion, the current study unveils the genomic evolution across the Correa cascade of intestinal-type gastric cancer, elucidates the underlying molecular mechanisms of gastric carcinogenesis, and provides evidence for potential personalized gastric cancer surveillance.

## Introduction

Gastric cancer remains one of the most prominent threats to public health worldwide, particularly in China and other East Asian countries. Over the past few decades, the diagnosis and treatment of its early-stage diseases in China have significantly improved, owing to advancements in cancer screening efforts. Early gastric cancer (EGC) accounts for approximately 20% of all diagnosed cases^[1]^. Accumulating guidelines for gastric cancer surveillance have been applied in clinical practice, mainly focusing on sex, age, family medical history, precancerous gastric lesions, unhealthy lifestyle, and *Helicobacter. pylori* (*H. pylori*) infection^[2–3]^. However, issues with both over-screening and under-screening for gastric cancer are present in China and other countries^[4]^. The feasibility of cancer screening relies heavily on the compliance of high-risk individuals, whereas the repeated screening of low-risk individuals significantly increases the financial burden on the healthcare system^[5]^. However, it is challenging to provide the participants with clear advice for subsequent gastroscopy when they are diagnosed with precancerous gastric lesions, because there is a lack of sufficient clinical evidence^[2]^. Therefore, elucidating the molecular mechanisms underlying gastric carcinogenesis and establishing more precise screening strategies are crucial for the efficacy of gastric cancer prevention, which may provide significant clinical and economic value.

Several studies have investigated the mechanisms of gastric carcinogenesis and identified a range of high-frequency gene mutations, including *TP53*, *ARID1A*, *CDH1*, *PIK3CA*, and *APC*^[6–8]^. Next-generation sequencing methods, including whole-genome sequencing, whole-exome sequencing, RNA sequencing, and targeted sequencing, have been used to describe molecular profiles and improve our understanding of the molecular mechanisms^[9–11]^. However, these studies have limitations, with sample collection being a key issue. Lauren intestinal type gastric adenocarcinoma, the most common type of gastric cancer, has been proposed to have a multistage histological evolution, starting from normal gastric mucosa, intestinal metaplasia (IM), low-grade intraepithelial neoplasia (LGIN), and high-grade intraepithelial neoplasia (HGIN) to cancer, which is defined as the Correa cascade^[12]^. Many of the aforementioned studies did not obtain sequential lesions from the same patients, which may have resulted in the findings being influenced by varying genetic backgrounds. Endoscopic submucosal dissection (ESD) has been commonly used in the treatment of EGC in East Asia and other areas^[13–14]^. Importantly, ESD-resected gastric specimens contain multiple stages of precancerous gastric lesions as well as EGC, which may potentially provide a source of sequential lesions^[15]^.

In the current study, we collected sequential lesions of the Correa cascade, including normal mucosa, IM, LGIN, HGIN, and EGC, in formalin-fixed and paraffin-embedded (FFPE) ESD-resected specimens from 14 Chinese patients by microdissection. With these samples, we performed whole-exome sequencing to determine the profiles of somatic aberrations during gastric carcinogenesis and validated the findings in a retrospective cohort of 39 patients with LGIN.

## Materials and methods

### Human gastric tissue specimens of EGC patients

**Figure 1:**
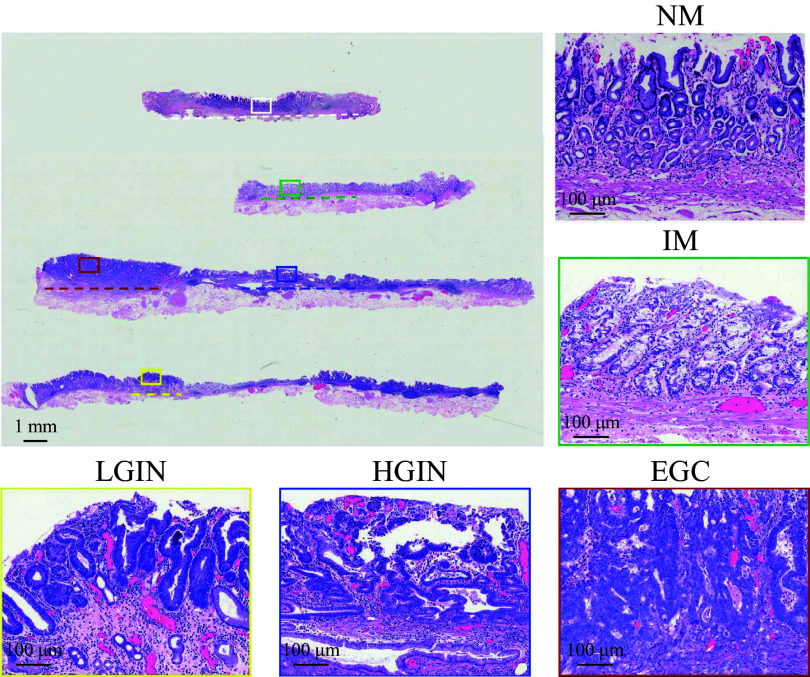
Collection of specimens of different Correa stages in the formalin-fixed and paraffin-embedded ESD-resected specimens of intestinal-type early gastric cancer using the microdissection technique. The slices of the ESD specimen in one patient were shown on the left. The dotted lines indicated lesion areas, and each square frame was shown in the right corresponding box. White box: NM; green box: IM; yellow box: LGIN; blue box: HGIN; red box: EGC. Abbreviations: ESD, endoscopic submucosal dissection; NM, normal gastric mucosa; IM, intestinal metaplasia; LGIN, low-grade intraepithelial neoplasia; HGIN, high-grade intraepithelial neoplasia; EGC, early gastric cancer.

To avoid the influence of tissue damage in FFPE specimens on sequencing, we only reviewed the pathological database of EGC patients who underwent complete ESD resection between January 2020 and July 2022 at the Affiliated Hospital of Nanjing University of Chinese Medicine. From these patients, we identified 14 patients with sequential lesions from IM to EGC in their FFPE specimens, who were then included in the sequencing group. The lesion areas of IM, LGIN, HGIN, and EGC were distinguished under a high-magnification microscope according to the World Health Organization (WHO) Classification of Gastric Carcinoma (5^th^ edition) (***Fig. 1***).

In the validation group, 39 patients diagnosed with LGIN by endoscopic biopsy were recruited between May 2015 and December 2019 at the same hospital. All these patients underwent ESD for potential early gastric malignancy during the follow-up, of whom 14 were diagnosed with HGIN or EGC, whereas the others were diagnosed with LGIN. There was no overlap in time or cases between the two groups.

To investigate the potential roles of IM in microsatellite instable (MSI) type gastric cancer, an additional 10 patients with MSI type EGC treated by ESD were enrolled. For WNT pathway activation analysis, we recruited an additional 10 cases of EGC and 10 cases of locally advanced gastric cancer (AGC) of the Lauren intestinal type for proof-of-concept assays of WNT pathway activation.

All clinicopathological data of the enrolled patients were collected, and all pathological characteristics were reviewed by two experienced endoscopists and two professional pathologists. All the samples were collected after obtaining informed consent from the enrolled patients following an established protocol approved by the Institutional Review Board of Nanjing University of Chinese Medicine. The current study was conducted in conformity with the principles of the Declaration of Helsinki.

### Preparation of pathological specimens using microdissection

The pathologists reviewed the preserved hematoxylin and eosin (H&E)-stained sections and evaluated the sizes of IM, LGIN, HGIN, and EGC. Each enrolled patient presented with four typical lesions greater than 2 mm in diameter, which is a prerequisite for obtaining sufficient DNA. First, ten 6-µm-thick slices were cut from the FFPE tissues. The slices were dewaxed in xylene, rehydrated in graded ethanol, and rinsed in 1× phosphate-buffered saline. Typical lesions were identified and marked precisely under the microscope. Next, these marked lesions were scraped accurately using a surgical blade microscopically and were collected into 1.5-mL Eppendorf tubes. Thus, each case had five tubes of specimens, namely normal gastric mucosa (NM), IM, LGIN, HGIN, and EGC (***Fig. 1***).

### Library preparation and sequencing

Genomic DNA from the FFPE ESD specimens was extracted with the QIAamp DNA FFPE Tissue Kit and DNeasy Blood and Tissue Kit (Qiagen, CA, USA), and quantified by Qubit 3.0, using the dsDNA HS Assay Kit (ThermoFisher Scientific, KS, USA). Normal gastric mucosa was used as the control. Library preparation was performed using a KAPA Hyper Prep Kit (KAPA Biosystems, MA, USA). Target enrichment was performed using the xGen Exome Research Panel and Hybridization and Wash Reagents Kit (Integrated DNA Technology, IA, USA) according to the manufacturer's protocol. The enriched libraries were amplified and purified by the on-bead PCR. Sequencing was performed on the Illumina HiSeq4000 platform using the PE150 sequencing chemistry (Illumina, CA, USA).

### Mutation calling

Trimmomatic^[16]^ was used for FASTQ file quality control. Low-quality leading/trailing (quality reading < 20) or undetermined bases were removed. Paired-end reads were then aligned to the reference human genome (build hg19) using the Burrows-Wheeler Aligner (BWA)^[17]^ with default parameters. PCR deduplication was performed using Picard (https://broadinstitute.github.io/picard/) and local realignment was performed around the indels. Base quality score recalibration was performed using GATK3 (https://software.broadinstitute.org/gatk/). Specifically, all the lesion samples, including IM, LGIN, HGIN, and EGC, were required to have pre-deduplication sequencing depths greater than 200× and post-deduplication depths greater than 60×. For adjacent normal control samples, the required sequencing depths were greater than 100× pre-deduplication and greater than 30× post-deduplication. Any samples that did not meet these quality control criteria were excluded from the analysis. The Picard tool was used to evaluate DNA damage. Samples with Total OScores below 35 were excluded. Single nucleotide variants and indels were identified using VarScan2^[18]^ with the minimum variant allele frequency threshold set at 2%. Variants were further filtered using the following parameters: (1) minimum read depth = 20; (2) minimum base quality = 15; (3) minimum variant supporting reads = 5; (4) variant supporting reads mapped to both strands; (5) strand bias not greater than 10%; (6) exclusion if present in more than 1% population frequency in the 1000 Genomes Project or East Asian populations from the Exome Aggregation Consortium (ExAC_ALL and ExAC_EAS) database; and (7) removed based on an internally collected list of recurrent sequencing errors using a normal pool of 100 samples.

The tumor mutational burden (TMB), chromosomal instability score (CIS), loss of heterozygosity (LOH), and intratumor heterogeneity (ITH) were determined as described in ***Supplementary Data*** (available online).

### Pathway analysis

We employed two pathway analysis methods to assess differences between groups, focusing solely on single nucleotide variants. First, we used the KEGG REST API (https://www.kegg.jp/kegg/rest/keggapi.html) to obtain the latest KEGG pathway gene annotations, which served as the reference for mapping genes. The enrichment analyses were conducted using the R package clusterProfiler (version 3.14.3). Enriched pathways with a *P*-value less than 0.01 were identified. In addition to the KEGG pathway analysis, we examined ten canonical oncogenic pathways, referencing insights from a previous study published in 2018^[19]^. A pathway was classified as abnormal if it contained one or more gene mutations. We then compared the differences in pathway abnormalities between groups using Fisher's exact test.

### Evolutionary analysis

To estimate the clonal correlations between lesions, Treeomics^[20]^ was used to reconstruct the evolutionary dynamics of early gastric cancer initiation. Treeomics uses a uniquely designed Bayesian inference model to account for the error-prone sequencing and varying low neoplastic cell content to calculate the probability that a specific variant is present or absent in each lesion. For each case, Treeomics was used to calculate the posterior probabilities of a variant being present based on total read depth and the number of reads covering the alternative allele. We used two indices to measure genetic heterogeneity: Jaccard similarity coefficients and genetic distances ('divergence'). The Jaccard similarity coefficient is defined as the ratio of shared variants to all variants (shared plus discordant) between samples. Genetic distance is defined as the total number of non-shared genetic variants present between two samples.

### Immunohistochemistry assay

Immunohistochemistry (IHC) assays were performed according to standard protocols. The monoclonal antibodies used were mouse anti-TP53 (1∶600, Cat. M7001, Dako, Carpinteria, USA), mouse anti-MLH1 (1∶200, Cat. #IR079, Dako), mouse anti-MSH2 (1∶200, Cat. #IR085, Dako), mouse anti-MSH6 (1∶200, Cat. #IR086, Dako), mouse anti-PMS2 (1∶200, Cat. #IR087, Dako), mouse anti-β-catenin (1∶200, Cat. #ZM-0442, ZSGB.BIO, Beijing, China), rabbit anti-PRKDC (1∶100, Cat. #ab32566, Abcam, Cambridge, UK), and rabbit anti-PCLO (1∶100, Cat. #ab307656, Abcam). If fewer than 50% of the cell nuclei were scattered or weakly to moderately positive for TP53, the sample was defined as wild-type. If more than 50% of the cell nuclei presented diffuse strong positivity for TP53 (missense mutation) or were completely unexpressed (nonsense mutation), it was classified as a mutant-type. Mismatch repair (MMR) proteins, including MLH1, PMS2, MSH2, and MSH6, are typically expressed in the nuclei of epithelial and stromal cells. If any one (at least one) of the four proteins lacked expression in the cells, it was defined as a defective mismatch repair (dMMR) phenotype. β-Catenin is commonly expressed in the cell membrane and cytoplasm, and the WNT pathway is activated when it transitions to the nucleus. PRKDC is mainly expressed in the nucleus and cytoplasm. The percentage of PRKDC-positive cells < 5%, 5%–25%, 25%–50%, and ≥ 50% was scored as 0, 1, 2, and 3, respectively. Color intensity was scored as 0, 1, and 2 points for no color, light yellow, and brown-yellow, respectively. If their sum was ≤ 3, PRKDC was negative; otherwise, the sample was considered PRKDC-positive.

### Statistical analysis

Normally distributed data were compared using Student's *t*-test, and non-normally distributed data were compared using the Wilcoxon rank-sum test. Categorical variables were compared using Fisher's exact test. Comparisons among multiple groups were performed using the Kruskal–Wallis test, followed by Dunn's multiple comparison test. Trend analysis was performed using Cuzick's test. The significance level was set at *P* < 0.05. All statistical analyses were performed using R software (version 3.5.2).

## Results

### Clinicopathologic analysis of the enrolled patients in the sequencing group

Fourteen patients with sequential lesions from IM to EGC were included in the sequencing group. Thirteen patients were male (13/14, 92.9%), and the median age was 69 years (range, 58–76 years) (***Table 1***). *H. pylori* was detected in approximately half of the patients (6/14, 42.86%) by hematoxylin and eosin (H&E) staining, indicating current *H. pylori* infection. Some patients had a history of smoking, alcohol consumption, high-salt diet, or exposure to pickled food, and two patients had a clear family medical history, with at least one case of gastric cancer among their first-degree relatives. Most lesions (9/14, 64.29%) were located in the upper third of the stomach.

**Table 1:** Clinicopathologic characteristics of the enrolled intestinal-type gastric cancer patients in the sequencing group

Patients	Age (years)	Sex	*H. pylori* infection	Smoking history	Alcohol history	Pickled/salted food intake	Family history of GC	Tumor location	Tumor stage	Lymphatic invasion	Microsatellite status	TP53 status
P01	73	M	Negative	No	No	No	No	M	SM2	Negative	MSS	MT
P02	73	M	Positive	No	No	No	No	L	SM1	Negative	MSI	WT
P03	59	M	Negative	No	No	No	No	U	SM2	Negative	MSS	WT
P04	70	M	Positive	No	Yes	No	No	U	SM1	Negative	MSS	MT
P05	69	M	Positive	No	No	No	No	U	SM2	Negative	MSS	MT
P06	60	M	Positive	No	No	No	Yes	U	SM2	Negative	MSS	MT
P07	76	M	Negative	Yes	No	No	Yes	U	SM2	Negative	MSS	WT
P08	64	M	Negative	No	No	Yes	No	U	SM1	Negative	MSS	MT
P09	58	M	Positive	Yes	Yes	Yes	No	U	SM2	Negative	MSS	WT
P10	75	F	Positive	No	No	No	No	U	SM2	Positive	MSS	MT
P11	65	M	Negative	Yes	Yes	Yes	No	M	SM1	Negative	MSS	MT
P12	74	M	Negative	No	No	No	No	L	SM1	Negative	MSS	WT
P13	69	M	Negative	No	No	Yes	No	L	SM1	Positive	MSS	WT
P14	67	M	Negative	Yes	Yes	No	No	U	SM1	Negative	MSS	MT
TP53 status: if less than 50% of cell nuclei were scattered or weakly to moderately positive for TP53, it was defined as wild-type (WT); if more than 50% of cell nuclei presented diffuse strong positive for TP53 (missense mutation) or completely unexpressed (nonsense mutation), it was mutant-type (MT).Abbreviations: GC, gastric cancer; F, female; M, male; U, upper 1/3 of the stomach; M, middle 1/3 of the stomach; L, lower 1/3 of the stomach; SM1, tumor infiltration confined in the superficial submucosal layer, less than 500 μm from the muscularis mucosae; SM2, tumor infiltration confined in the deep submucosal layer, more than 500 μm from the muscularis mucosae; MSI, microsatellite instable; MSS, microsatellite stable.												

A total of 56 samples were obtained from 14 patients at different stages (*i.e.*, IM, LGIN, HGIN, and EGC), along with 14 matched normal control samples (NM). All the samples passed depth quality control. The mean sequencing depths were 503× pre-deduplication and 139× post-deduplication for all lesion samples, and 393× pre-deduplication and 108× post-deduplication for NM. In addition, all samples passed deamination damage/oxidative damage contamination quality control analysis for the FFPE samples, qualifying them for further analysis.

### Genetic landscape in gastric carcinogenesis

**Figure 2:**
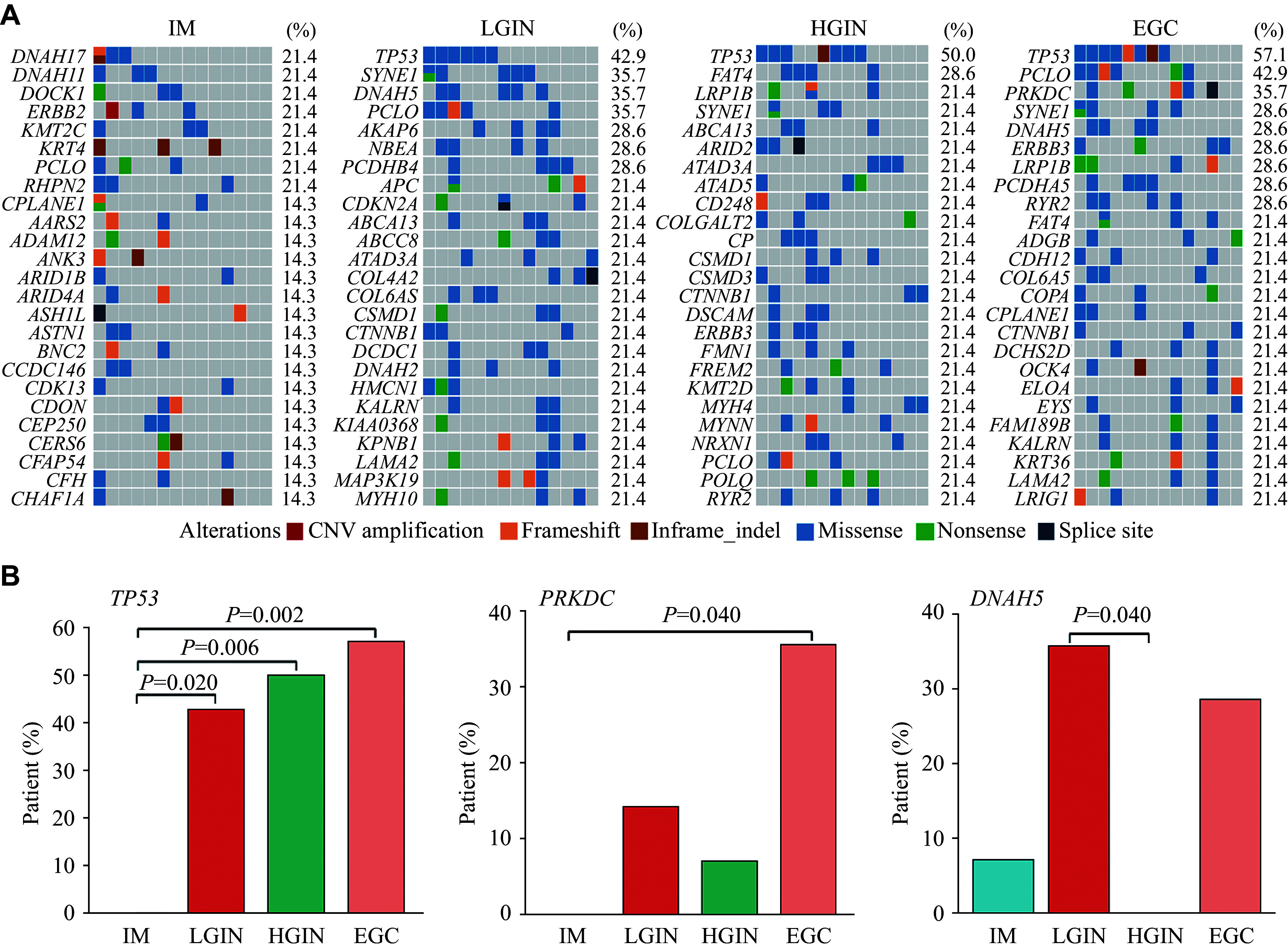
Genetic landscape of gastric carcinogenesis. A: Oncoplot showing the most frequently mutated genes in IM, LGIN, HGIN, and EGC samples (*n* = 14). The top 25 recurrently mutated genes in each stage were included in the analysis. B: Comparisons of mutational frequencies of *TP53*, *PRKDC*, and *DNAH5* across IM, LGIN, HGIN, and EGC samples (*n* = 14). *P*-value by the Wilcoxon rank-sum test. Abbreviations: EGC, early gastric cancer; IM, intestinal metaplasia; LGIN, low-grade intraepithelial neoplasia; HGIN, high-grade intraepithelial neoplasia; CNV, copy number variation.

To determine the molecular events underlying gastric cancer evolution, we profiled the samples across the Correa cascade, and included the top 25 recurrently mutated genes at each stage in this analysis (***Fig. 2A***). In EGC, the most frequently mutated genes were *TP53* (57.1%), *PCLO* (42.9%), and *PRKDC* (35.7%), but no *TP53* or *PRKDC* mutations were detected in IM. Importantly, we found a high frequency of *TP53* alterations (42.9%; *vs.* IM, *P* = 0.020) in LGIN, which further increased in HGIN (50.0%; *vs.* IM, *P* = 0.006) and EGC (57.1%; *vs.* IM, *P* = 0.002) (***Fig. 2B***). No significant difference in the frequency of *TP53* alterations was found among these three stages (*i.e.*, LGIN, HGIN, and EGC). While *PRKDC* alterations were detected in LGIN, HGIN, and EGC, only EGC showed a significant enrichment in *PRKDC* alterations, compared with IM (35.7% *vs.* 0%, *P* = 0.040; ***Fig. 2B***). Except for HGIN, we observed *DNAH5* mutations in all other Correa stages. LGIN exhibited the highest frequency of *DNAH5* mutations, which was significantly different from HGIN (35.7% *vs.* 0%, *P* = 0.040; ***Fig. 2B***). Comparisons of gene mutation frequencies across the four Correa stages are shown in ***Supplementary Fig. 1*** (available online).

Given the potential biological relevance of *APC* in gastric cancer development, we analyzed its mutational frequency at different stages. *APC* was mutated in 7.1%, 28.6%, 14.3%, and 14.3% of IM, LGIN, HGIN, and EGC lesions, respectively (***Supplementary Fig. 2***, available online). Although loss-of-function mutations in *APC* were detected as early as in LGIN, no clear association with the disease stage was noted.

We also examined intergroup copy number variation (CNV) levels at the single-gene level (***Supplementary Fig. 3***, available online). While EGC appeared to show a trend of increased CNV events, no difference was found between the four Correa stages.

### Genomic similarities of IM, LGIN, HGIN, and EGC

**Figure 3:**
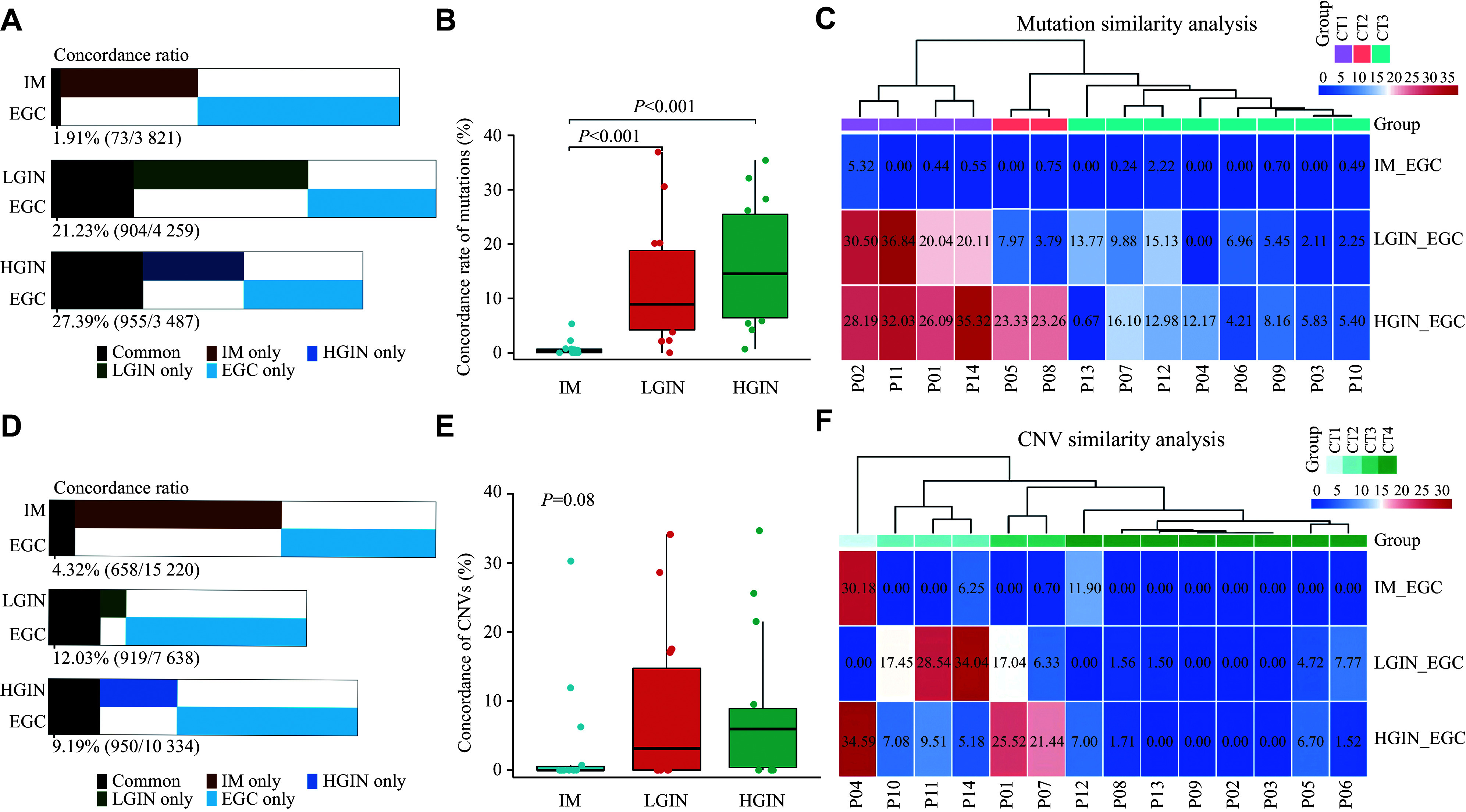
Overall similarities at the mutation and CNV levels. A: Percentages of shared mutations in IM, LGIN, and HGIN with EGC (*n* = 14). B: Statistical analysis of the concordance ratios of mutations in IM, LGIN, and HGIN with EGC (*n* = 14). C: Clustering of case-by-case concordance ratio with EGC at the mutation level. A concordance ratio threshold of ≥ 20% is defined as "similar". Cases are categorized into three tiers: CT1 (highly similar) for cases with similarities in two or more stages compared with EGC (*n* = 4); CT2 (moderately similar) for cases with similarities in only one stage compared with EGC (*n* = 2); and CT3 (low similarity) for cases where all stages have a concordance ratio below the threshold (*n* = 8). D: Percentages of shared CNV events in IM, LGIN, and HGIN with EGC (*n* = 14). E: Statistical analysis of the concordance ratios of CNV events in IM, LGIN, and HGIN with EGC (*n* = 14). F: Clustering of case-by-case concordance ratio with EGC at the CNV level. *P*-value by the Wilcoxon rank-sum test. Abbreviations: EGC, early gastric cancer; IM, intestinal metaplasia; LGIN, low-grade intraepithelial neoplasia; HGIN, high-grade intraepithelial neoplasia.

Next, we analyzed genomic similarities during the evolution from IM to EGC. In total, we detected 1573 variants in IM, 2842 in LGIN, 2121 in HGIN, and 2321 in EGC. The concordance ratio with EGC showed an increasing trend across the different stages of EGC, with IM sharing 1.91% of the variants (73 of 3821 total), LGIN sharing 21.23% (904 of 4259), and HGIN sharing 27.39% (955 of 3487; ***Fig. 3A***). All the samples were characterized by a high prevalence of C>T transitions (***Supplementary Fig. 4A***, available online), with predominantly mutational signatures of aging (***Supplementary Fig. 4B***, available online). The matched sample analysis revealed some notable differences in the concordance ratio of EGC between the different groups. Specifically, both LGIN and HGIN showed a higher concordance ratio with EGC than IM (*P* < 0.001), with no difference observed between LGIN and HGIN (***Fig. 3B***). Hierarchical clustering based on the concordance ratio revealed mutational similarity in 42.9% of patients (6 of 14 patients; ***Fig. 3C***). Of these patients, we observed that both LGIN and HGIN showed a high similarity with EGC in four patients (*i.e.*, P02, P11, P01, and P14). In addition, only HGIN showed a notable similarity with EGC in two patients (P05 and P08). In the remaining eight patients, a low concordance ratio was observed across the IM, LGIN, and HGIN groups. Interestingly, one case (P02) was determined as having MSI-type EGC (***Supplementary Fig. 4B***), and the IHC assay indicated that MutL homolog 1 (MLH1) and postmeiotic segregation increased 2 (PMS2) were deficient in LGIN, HGIN, and EGC lesions, while they were detected in the IM area (***Supplementary Fig. 5***, available online).

We also analyzed the similarities in CNV events across different stages. We detected 9050 CNV events in IM, 1729 in LGIN, 4456 in HGIN, and 6828 in EGC. Despite the high number of CNV events in IM, the concordance ratio with EGC was only 4.32% (658 of 15220; ***Fig. 3D***). In contrast, the concordance ratios in LGIN and HGIN were 12.03% (919 of 7638) and 9.19% (950 of 10334), respectively. While no significant difference in the concordance ratio was observed among the three groups (*P* = 0.076), IM showed a trend towards lower concordance than LGIN (*P* = 0.200) and HGIN (*P* = 0.100), respectively (***Fig. 3E***). Consistent with the case-by-case mutational similarity analysis, both LGIN and HGIN in P01 showed high concordance with EGC in terms of CNV events (***Fig. 3F***). P11 and P14 also showed CNV events similar to those in LGIN and EGC. In addition, P10 was found in the same cluster as P11 and P14, whereas P07 showed CNV events similar to those in HGIN and EGC. A low concordance ratio for CNV events was observed across IM, LGIN, and HGIN in the remaining patients.

Furthermore, we compared the concordance ratio as assessed at both the mutation and CNV levels, which revealed that IM showed a lower degree of similarity to EGC than LGIN and HGIN at both the mutation and CNV levels (***Supplementary Fig. 6***, available online). Although no significant difference in concordance ratios was observed between the mutation and CNV assessments in both the IM and LGIN groups, HGIN showed a higher level of mutational similarity than CNV (*P* = 0.040). Notably, one subject with MSI (P02) showed a high TMB in the IM (12.86 muts/Mb). In this case, IM showed a high concordance ratio with EGC (5.32%) at the mutation level, which was significantly higher than the median concordance ratio (0.34%) of IM samples from other subjects. In addition, the mutational signatures of all four samples from this patient showed a high prevalence of MMR deficiencies (***Supplementary Fig. 4B***). As patients with MSI may develop malignancies^[21]^, these results indicate that IM states may provide some useful information for screening patients with MSI. We further performed IHC assays in an additional 10 cases of EGC with MSI, and found that MLH1 and PMS2 were preserved in IM areas, but we did not find them in LGIN, HGIN, and EGC lesions, which was consistent with P02.

### Associated pathways and genomic characterization

**Figure 4:**
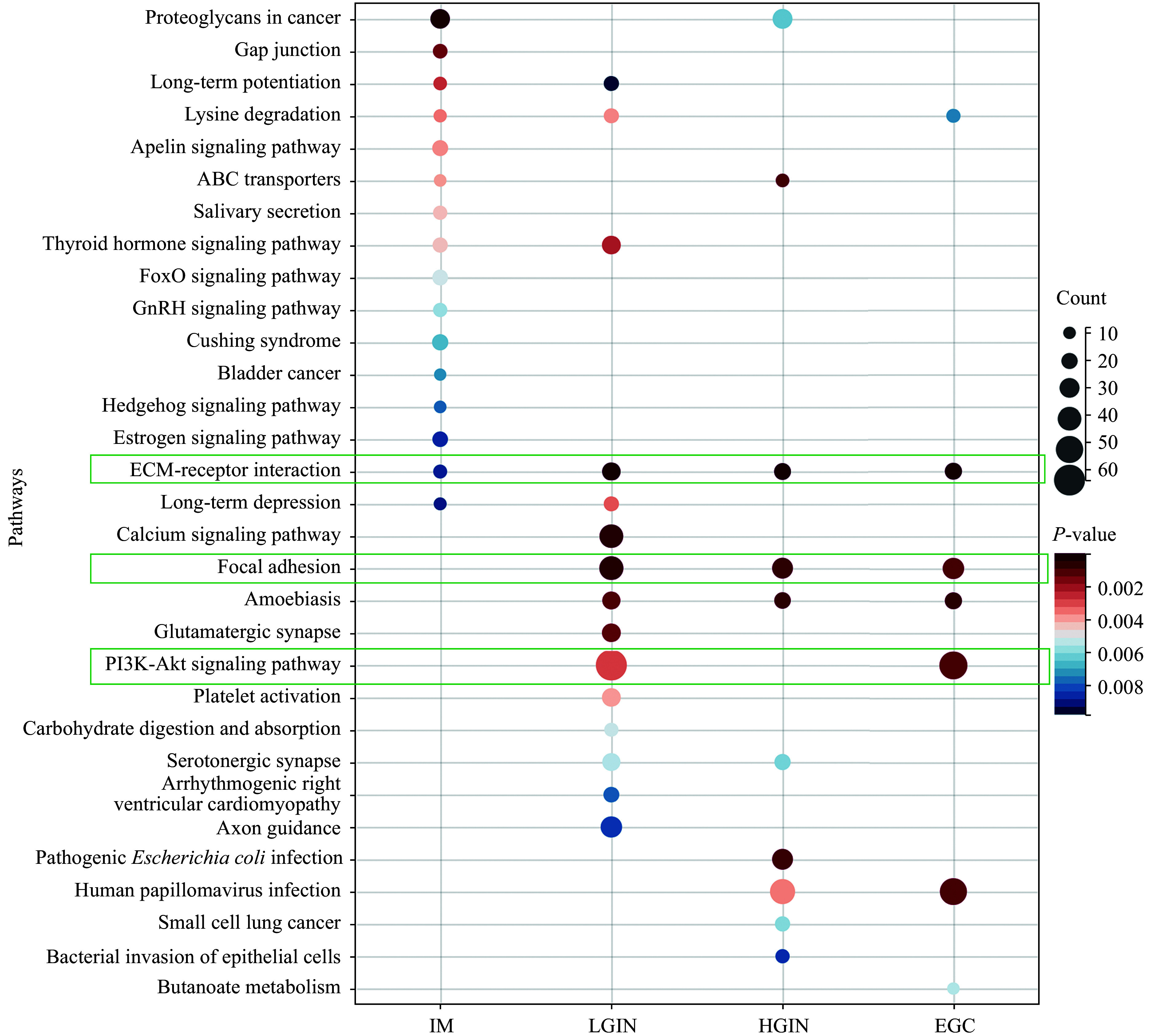
Pathways associated with early gastric cancer development. KEGG signaling pathway enrichment analysis was conducted for alterations at each Correa stage. There were 16, 15, 10, and seven signaling pathways enriched in IM, LGIN, HGIN, and EGC, respectively. Three signaling pathways involved in tumorigenesis and development, including ECM-receptor interaction, focal adhesion, and PI3K-Akt pathways, were identified in EGC. Abbreviations: EGC, early gastric cancer; IM, intestinal metaplasia; LGIN, low-grade intraepithelial neoplasia; HGIN, high-grade intraepithelial neoplasia.

We then conducted KEGG signaling pathway enrichment analysis for alterations at each Correa stage. IM, LGIN, HGIN, and EGC were enriched in 16, 15, 10, and seven signaling pathways, respectively. Three signaling pathways involved in carcinogenesis and development were identified in EGC, including the extracellular matrix (ECM)-receptor interaction, focal adhesion, and PI3K-Akt pathways (***Fig. 4***). Notably, the IM stage predominantly showed a unique set of signaling pathways, suggesting its divergence from other stages (***Fig. 4***). Considering the signaling pathways enriched in EGC, the precancerous stages IM, LGIN, and HGIN shared two (28.6%), five (71.4%), and four (57.1%) signaling pathways with EGC, respectively, suggesting a potential progression towards gastric cancer.

**Figure 5:**
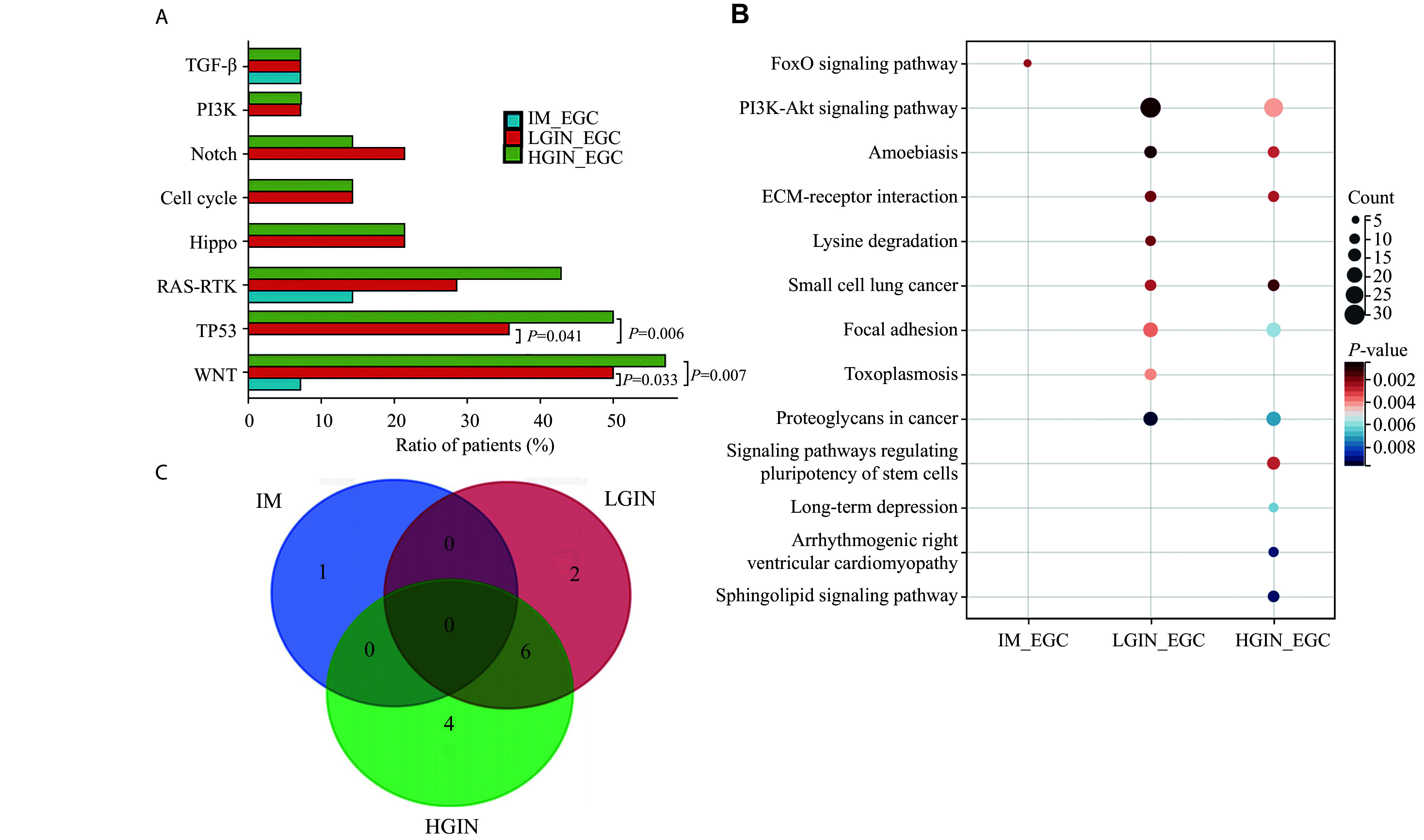
The canonical oncogenic signaling pathways involved in early gastric cancer development. A: Enrichment of genetic alterations shared with EGC in 10 canonical oncogenic signaling pathways. TP53 pathway gene alterations occurred late in the transition from IM to EGC and were detected only in LGIN, HGIN, and EGC samples. WNT pathway gene alterations showed a significant increase from the initial IM to LGIN and continued to increase during disease progression (*n* = 14). B: KEGG analysis showing pathway enrichment of shared genetic alterations with EGC (*n* = 14). C: Overlap of enriched pathways among IM, LGIN, and HGIN that were shared with EGC (*n* = 14). *P*-value by the Wilcoxon rank-sum test. Abbreviations: EGC, early gastric cancer; IM, intestinal metaplasia; LGIN, low-grade intraepithelial neoplasia; HGIN, high-grade intraepithelial neoplasia.

We further evaluated the genetic alterations shared with EGC in 10 canonical oncogenic signaling pathways^[19]^. Consistent with previous reports^[22–23]^, genetic alterations shared with EGC were enriched in WNT, TP53, RAS-RTK, and Hippo pathway genes (***Fig. 5A***). Few shared oncogenic pathway gene alterations were detected in IM. In particular, TP53 pathway gene alterations occurred late in the transition from IM to EGC and were detected only in LGIN, HGIN, and EGC samples (LGIN *vs.* IM, *P* = 0.041; HGIN *vs.* IM, *P* = 0.006). In contrast, WNT and RAS-RTK pathway gene alterations were detected as early as in IM (***Fig. 5A***). Alterations in the RAS-RTK pathway genes increased incrementally, while alterations in the WNT pathway genes showed a significant increase from the initial IM to LGIN and continued to increase during disease progression (LGIN *vs.* IM, *P* = 0.033; HGIN *vs.* IM, *P* = 0.007; ***Fig. 5A***). However, our proof-of-concept study with 10 cases of EGC and 10 cases of AGC showed that β-catenin was strongly expressed in the cytoplasm of IM, LGIN, HGIN, cancer, and normal mucosa in nine cases (four EGCs and five AGCs), and 10 cases presented decreased β-catenin expression in neoplastic areas compared with normal mucosa (six EGCs and four AGCs). Only one case of AGC showed positive expression in the nucleus of neoplastic areas, indicating that the WNT pathway is activated in only a minority of gastric cancers (***Supplementary Fig. 7***, available online).

Subsequent KEGG analysis revealed that EGC-shared alterations were enriched in only one pathway in the IM stage, but were present in eight and 10 signaling pathways in LGIN and HGIN, respectively (***Fig. 5B*** and ***5C***). The enrichment of oncogenic pathways, such as focal adhesion, ECM-receptor interaction, and PI3K-Akt, was observed in LGIN and HGIN, further confirming their potential involvement in the early stages of gastric cancer initiation.

**Figure 6:**
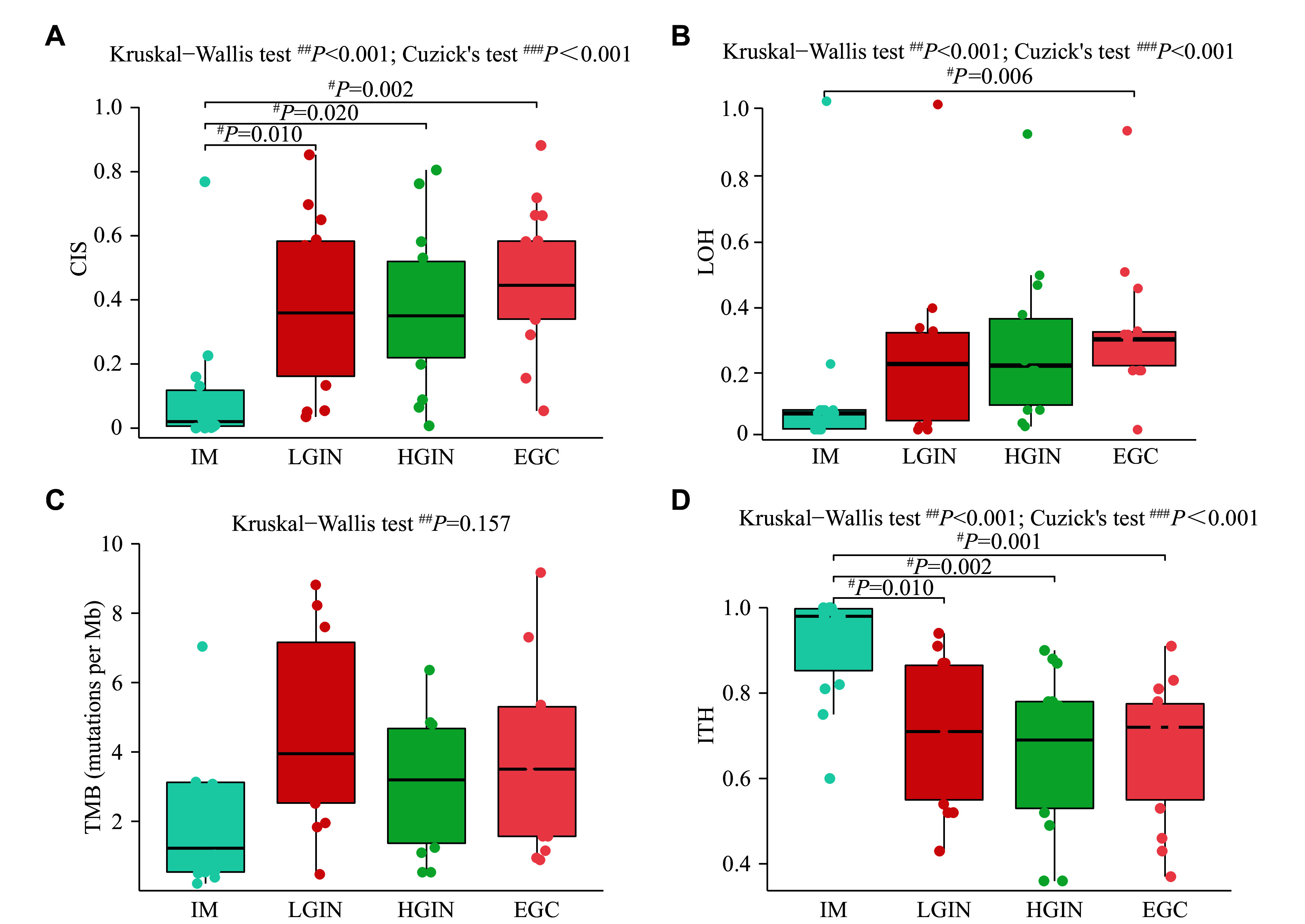
Genomic characteristics of gastric carcinogenesis. A–D. Comparisons of (A) chromosomal instability score (CIS), (B) loss of heterozygosity (LOH), (C) tumor mutational burden (TMB), and (D) intratumor heterogeneity (ITH) among IM, LGIN, HGIN, and EGC. *n* = 14. ^#^*P*-value by the Wilcoxon rank-sum test. ^##^*P*-value by Kruskal–Wallis test followed by Dunn's multiple comparisons test.^ ###^*P*-value by Cuzick's test for trend analysis. Abbreviations: EGC, early gastric cancer; IM, intestinal metaplasia; LGIN, low-grade intraepithelial neoplasia; HGIN, high-grade intraepithelial neoplasia.

Chromosomal instability and LOH have also been implicated in gastric cancer development^[24]^. Indeed, increasing trends in the CIS (Cuzick's test, *P* < 0.001; ***Fig. 6A***) and LOH (Cuzick's test, *P* < 0.001; ***Fig. 6B***) were observed during the IM-to-EGC transition. CIS levels increased significantly from IM to LGIN, with the latter three stages showing much higher CIS levels in LGIN than in IM (LGIN, *P* = 0.010; HGIN, *P* = 0.020; EGC, *P* = 0.002). EGC also showed a higher level of LOH compared with IM (*P* = 0.006). In addition, we assessed TMB, and no association with disease stage was found (*P* = 0.157, ***Fig. 6C***). Interestingly, ITH further decreased with gastric cancer evolution (Cuzick's test, *P* < 0.001), with IM showing the highest level of ITH, compared with LGIN (*P* = 0.010), HGIN (*P* = 0.002), and EGC (*P* = 0.001, ***Fig. 6D***).

### Reconstruction of evolutionary dynamics

**Figure 7:**
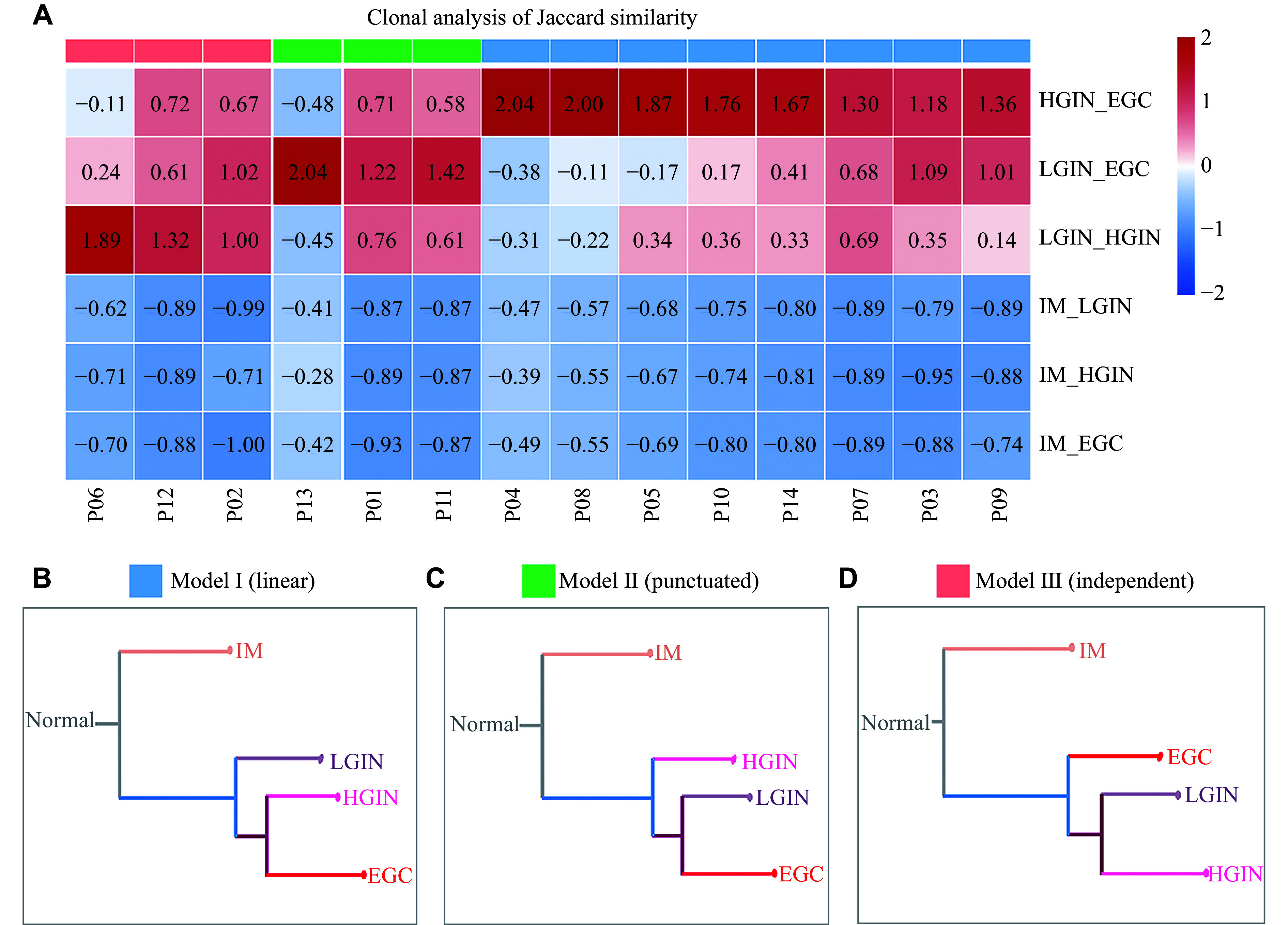
Reconstruction of the evolutionary process in early gastric cancer. A: Clustering based on the Jaccard similarity coefficient from pair-wise comparisons of IM, LGIN, HGIN, and EGC. B–D: Phylogenetic trees illustrating modes of evolution in early gastric cancer. The model Ⅰ (B), a linear progression from LGIN, to HGIN and then to EGC; the model Ⅱ (C), a punctuated evolution with LGIN directly progressing to EGC; the model Ⅲ (D), an independent evolution of EGC of LGIN/HGIN. Abbreviations: EGC, early gastric cancer; IM, intestinal metaplasia; LGIN, low-grade intraepithelial neoplasia; HGIN, high-grade intraepithelial neoplasia.

To reconstruct the evolutionary dynamics of early gastric cancer, we used Treeomics^[19]^ to quantify the Jaccard similarity coefficients between all pairs of samples in each patient. In all patients, the IM samples were highly dissimilar to those of LGIN, HGIN, and EGC (***Fig. 7A***). Clustering based on Jaccard similarity coefficients revealed three evolutionary trajectory models in these 14 patients. In model Ⅰ (linear), the majority of patients (8/14) showed a linear progression from LGIN to HGIN and finally to EGC. HGIN showed the highest similarity to EGC (***Fig. 7A*** and ***7B***). In model Ⅱ (punctuated), three of these patients (3/14), EGC showed a greater similarity to LGIN than to HGIN (***Fig. 7A***), resembling the mode of punctuated evolution, in which tumor cells were pre-programmed as early as LGIN (***Fig. 7C***). In model Ⅲ (independent), pairwise comparisons identified three cases (3/14) where LGIN and HGIN were the most closely related (***Fig. 7A***). In these patients, both LGIN and HGIN showed a limited similarity to EGC, suggesting an independent evolution of EGC (***Fig. 7D***). The case-by-case analyses of evolutionary trajectory are shown in ***Supplementary Fig. 8*** (available online).

**Figure 8:**
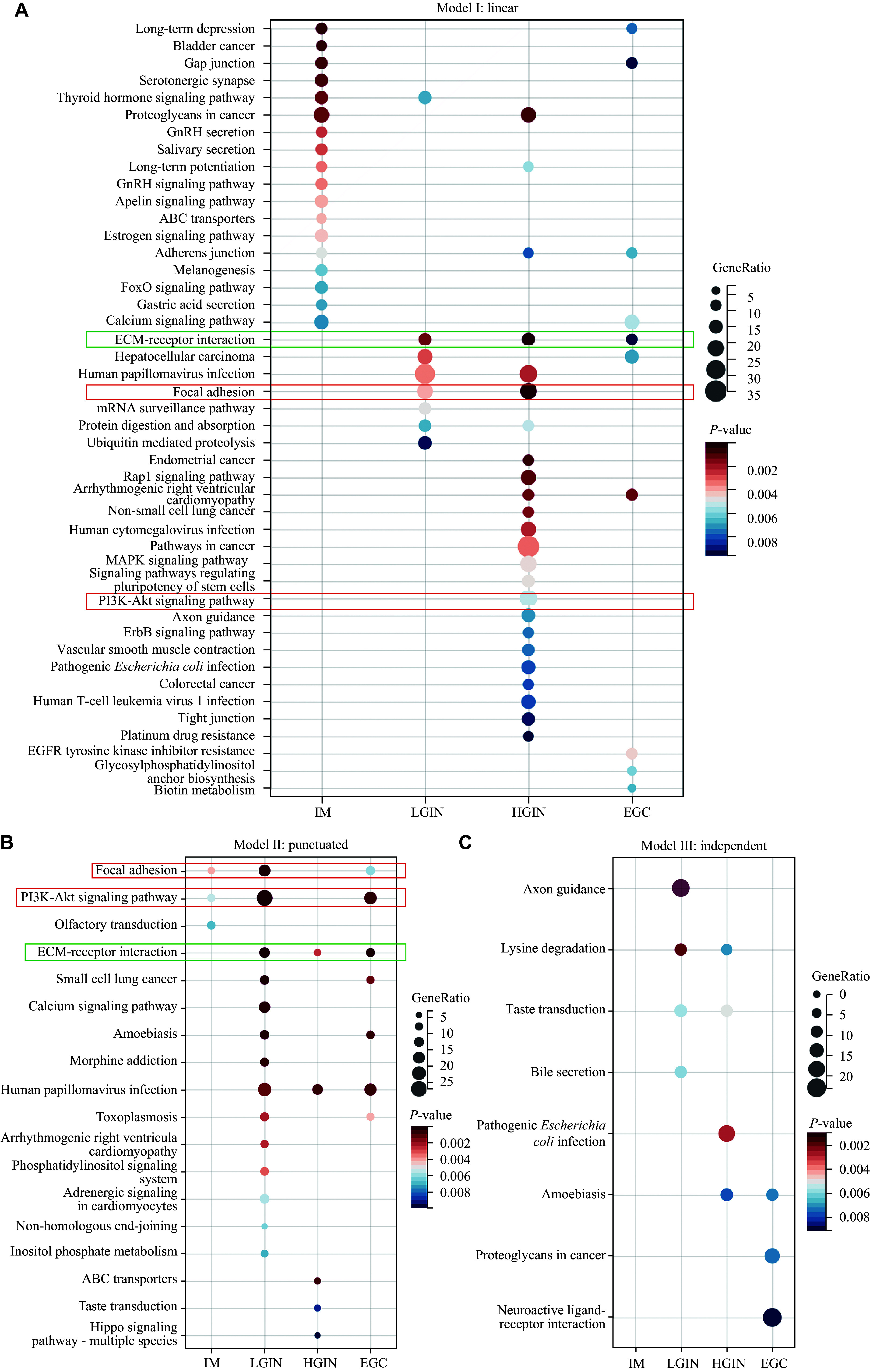
The dysregulated pathways involved in each model of evolution. The associations of dysregulated pathways with Correa stages in each of the evolution models were analyzed. A: In the linear evolution, the ECM-receptor interaction pathway showed enrichment across LGIN, HGIN, and EGC stages; B: The focal adhesion and PI3K-Akt pathways were involved in the punctuated model; C: None of these pathways were observed in the independent model. Abbreviations: EGC, early gastric cancer; IM, intestinal metaplasia; LGIN, low-grade intraepithelial neoplasia; HGIN, high-grade intraepithelial neoplasia.

We further investigated the associations of genetic mutations or dysregulated pathways with Correa stages in each of the evolution models. In the linear model, *TP53* mutations were detected in 50% of the samples across LGIN, HGIN, and EGC, but were absent in IM. However, these differences were not statistically significant. Similarly, no correlation was found between *TP53* mutations and the Correa stage in either punctuated or independent models (***Supplementary Table 1***, available online). At the signaling pathway level, we focused our analysis on the ECM-receptor interaction, focal adhesion, and PI3K-Akt pathways, which were prevalent in all EGC samples. In the linear model, these three pathways were prominent at different stages, particularly in HGIN (***Fig. 8A***). Among them, only the ECM-receptor interaction pathway showed enrichment across LGIN, HGIN, and EGC stages, suggesting a potentially higher dependency of linear evolution on this specific pathway. In contrast, in the punctuated model, both LGIN and EGC were enriched in all three pathways. However, the ECM-receptor interaction pathway was also enriched in HGIN, suggesting that the punctuated model relies more on focal adhesion and PI3K-Akt pathways (***Fig. 8B***). Notably, none of these pathways were observed in the independent model (***Fig. 8C***). Finally, no clear associations of these pathways with modes of evolution were found in the six patients with *H. pylori* infection (*i.e.*, P02, 04, 05, 06, 09, and 10) (*P* = 0.370, ***Supplementary Table 2***, available online).

### Validation of predictive value of *TP53*, *PCLO*, and *PRKDC* in LGIN progression to EGC

The results presented above indicate that LGIN may represent a true precancerous lesion for intestinal-type EGC. However, to date, there is no established marker for assessing the risk of LGIN progression to cancer. To address this, we performed a preliminary validation study using a retrospective cohort of 39 patients initially diagnosed with LGIN. Among them, 14 patients (subgroup #1) experienced progression to HGIN or EGC during follow-up, while the others (subgroup #2) remained diagnosed with LGIN (***Supplementary Table 3***, available online). There was no significant difference in age, sex, duration of follow-up, lesion size, or *H. pylori* infection status between these two subgroups (***Table 2***). We further examined the mutation status of *TP53*, *PCLO*, and *PRKDC* in these specimens. *TP53* or *PRKDC* mutations in subgroup #1 were 42.90% and 50.00%, respectively, which were significantly higher than those in subgroup #2 (*P* = 0.016 for *TP53*; *P* = 0.005 for *PRKDC*) (***Table 2***, ***Supplementary Fig. 9*** [available online]). In contrast, the mutation rate of *PCLO* was not significantly different between the two subgroups (*P* = 0.225). The sensitivity and specificity of *TP53* and *PRKDC* for predicting progression are shown in ***Table 3***. These results indicate that *PRKDC* mutations, in addition to *TP53* mutations, may serve as predictive markers for the risk of LGIN progression to HGIN or EGC.

**Table 2:** Analysis of clinicopathological characteristics in the validation group

Variables	Subgroup #1 (*n*=14)	Subgroup #2 (*n*=25)	*P*-value
Male [*n* (%)]	10 (71.4)	12 (48.0)	0.157
Age (first diagnosis of LGIN) (years, mean±SEM)	63.4±6.6	61.0±9.2	0.407
Duration of follow-up (months, mean±SEM)	34.9±20.1	24.7±14.3	0.072
Lesion location^a^ [*n* (%)]	5 (35.7)	18 (72.0)	0.036
Lesion size (mm, mean±SEM)	15.2±10.8	10.2±8.4	0.117
*H. pylori* positive infection [*n* (%)]	4 (28.6)	5 (20.0)	0.831
*TP53* mutant-type [*n* (%)]	6 (42.9)	2 (8.0)	0.016
*PRKDC* positive [*n* (%)]	7 (50.0)	2 (8.0)	0.005
*PCLO* positive [*n* (%)]	4 (28.6)	3 (12.0)	0.225
Subgroup #1: the lesions of LGIN in these 14 patients were aggravated into HGIN or EGC during the follow-up.Subgroup #2: the lesions of LGIN in these 25 patients retained the diagnosis of LGIN during the follow-up.^a^Lesion location indicates a case with the lower 1/3 of the stomach.Abbreviation: SEM, standard error of the mean.			

**Table 3:** The predictive value of *TP53* and *PRKDC* mutations for the progression of LGIN to HGIN or EGC

Genes	Sensitivity	Specificity
*TP53*	42.9% (6/14)	92.0% (23/25)
*PRKDC*	50.0% (7/14)	92.0% (23/25)
*TP53* or *PRKDC*	71.4% (10/14)	84.0% (21/25)

## Discussion

As one of the most prevalent malignancies, gastric cancer is often diagnosed at an advanced stage, resulting in prolonged suffering, aggressive treatments, and poor prognosis for most patients. A meta-analysis has indicated that endoscopic screening may reduce gastric cancer mortality, but not affect its incidence^[25]^, implying deficiencies in current screening strategies. Therefore, the objective of precision surveillance should shift from reducing mortality to preventing the development of gastric cancer. Most patients are diagnosed with Lauren intestinal-type gastric cancer. The application of next-generation sequencing may be superior in ascertaining genomic alterations at each Correa stage and determining potential drivers. Certainly, specimens of sequential lesions of the Correa cascade were crucial for this sequencing study. In the current study, we showed that ESD-resected specimens were advantageous for simultaneously providing sequential lesions of the Correa cascade. Furthermore, a reliable microdissection technique for FFPE specimens, guided by experienced pathologists, helped to isolate histologically identical cells while minimizing contamination from surrounding tissues. To the best of our knowledge, this is the first high-throughput sequencing study utilizing synchronously collected lesions at each stage of the Correa cascade, providing novel insight into the molecular evolution of early gastric cancer.

In the current study, we identified multiple alterations at each stage of early gastric carcinogenesis, of which *TP53*, *PCLO*, and *PRKDC* were the most frequently mutated genes in EGC (> 30% of patients), but no mutated *TP53* or *PRKDC* was detected at the IM stage. Of these genes, only *TP53* mutations were consistently present in LGIN, HGIN, and EGC, suggesting that *TP53* mutations may be an early event in the molecular evolution of intestinal-type EGC. These findings are consistent with previous findings. For example, in a 10-year prospective study, Huang *et al*^[26]^ reported that IM showed recurrent mutations in *FBXW7* instead of *TP53*. In contrast, Yoshida *et al*^[15]^ demonstrated that *TP53* was the most frequent mutated gene in well-differentiated intramucosal gastric cancers. Several studies have demonstrated that *TP53* mutations precede other genetic mutations in intestinal-type EGC^[27–28]^. As one of the most frequently mutated genes in gastric cancer^[6]^, *TP53* is significantly associated with high metastatic potential^[29]^. Multiple genomic classification systems for gastric cancer have included *TP53* status in their classification criteria^[30–32]^. Several studies have investigated the mechanisms underlying *TP53* mutations in gastric carcinogenesis. For example, Shimizu *et al*^[33]^ demonstrated that *H. pylori* infection increased cytidine deaminase activity in the gastric mucosa to promote the accumulation of *TP53* mutations. Similarly, Sethi *et al*^[34]^ reported that *TP53* inactivation enhanced the effects of environmental carcinogens to promote gastric premalignancy in murine model. However, to date, there is no clinical evidence linking *TP53* mutations to the development of EGC. Our findings provide additional support for the early involvement of *TP53* in the Correa cascade and highlight its potential as a marker for progression risk, particularly in LGIN.

Similarity analyses, including gene mutation or CNV profiling, involved pathways, chromosome instability or loss of heterozygosity, and the evolutionary model, indicated that the IM stage showed dissimilarity to the following LGIN, HGIN, and EGC stages, suggesting that IM is an independent stage. IM has also been proposed as a precursor to gastric cancer. A population-based study in North America showed that gastric IM is a predictor of gastric adenocarcinoma, with an incidence of gastric cancer of 0.72/1000 person-years, and the median time from IM to cancer was 6.1 years^[35]^. A meta-analysis also reported the association between incomplete IM or IM in the corpus and gastric cancer^[36]^. However, a recent study indicates that the presence of IM alone may not be sufficient to suggest an increased risk of gastric cancer^[37]^. Therefore, the genetic association between IM and EGC needs further investigation. Notably, we found that one patient with MSI had a high TMB at the IM stage, and that IM showed a significantly higher concordance ratio of gene mutations to EGC than the median concordance ratio. Further analysis of an additional 10 cases with MSI showed that MLH1 and PMS2 were preserved in IM areas, but were not detected in LGIN, HGIN, and EGC lesions. Such novel markers need to be investigated.

Three main signaling pathways, the ECM-receptor interaction, focal adhesion, and PI3K-Akt pathways, have been identified in intestinal-type gastric carcinogenesis. Together with analyses of 10 canonical oncogenic signaling pathways shared with EGC, we further revealed the associations of LGIN or HGIN, rather than IM, with EGC. Although gene alterations in the WNT pathway increased during disease progression, further examination did not reveal similar trends, which may be due to discrepancies between gene and protein expression. However, there was no evidence for the existence of a unique pathway for each stage, although the IM stage predominantly showed a unique set of signaling pathways, compared with the other stages.

Furthermore, we reconstructed the evolutionary dynamics of EGC and proposed three evolutionary models for intestinal-type EGC. The model Ⅰ, the main model, showed linear progression from LGIN to HGIN, resulting in EGC. The model Ⅱ showed a punctuated evolution from LGIN to EGC, whereas the model Ⅲ showed an independent evolution of EGC. Importantly, we analyzed the potential pathways involved in each model. The ECM-receptor interaction pathway may be the driver in the linear model, whereas the focal adhesion and PI3K-Akt pathways may be involved in the punctuated model. However, no definite pathway was observed in the independent model, suggesting that gastric cancer in model Ⅲ may have distinct drivers. Although *H. pylori* infection has been shown to be involved in intestinal-type gastric carcinogenesis, no association has been found between *H. pylori* status and evolutionary models.

The current study indicates that LGIN possesses molecular characteristics similar to those of cancer and should be a veritable premalignant lesion for EGC, and LGIN has been shown to have a risk of progression to EGC. However, the evaluation of the risk of LGIN progression to cancer remains unclear. Chen *et al*^[38]^ constructed a predictive model based on sex, multi-site involvement, hyperemia, ulcer, and morphology to determine the risk. Additionally, our retrospective validation showed for the first time that *PRKDC* mutations, in addition to *TP53* status, may be used for risk stratification in LGIN. LGIN with *PRKDC* or *TP53* mutations has a higher risk of progression to gastric cancer than LGIN without these mutations. Therefore, we proposed that regular endoscopic surveillance should be recommended for higher-risk patients, and timely treatment of high-risk LGIN may potentially reduce the incidence of gastric cancer. Further prospective studies with larger cohorts are crucial for establishing the predictive value of these mutations, which is of great importance for gastric cancer prevention.

However, the current study had some limitations. The number of patients enrolled was relatively small because of the difficulty in obtaining clinical samples. Although synchronous lesions of the Correa cascade were examined in the current study, the analysis of sequential metachronous lesions of the Correa cascade during longitudinal surveillance may provide more useful insights. Future prospective studies should aim to identify suitable specimens for such molecular evolution studies. In addition, whole exome sequencing has a relatively shallow sequencing depth. We set a higher threshold for mutation allele fraction and read counts to minimize false-positive results, which may lead to the missed detection of some low-frequency mutations, especially at the IM stage.

In summary, the current study revealed the molecular evolution according to the Correa cascade in intestinal-type gastric cancer. The results highlight the high heterogeneity and complexity of carcinogenesis in intestinal-type gastric cancer and provide some novel evidence for potential markers that could be used in the precision prevention of gastric cancer, which may potentially lead to a reduction in its incidence in the near future.

## SUPPLEMENTARY DATA

Supplementary data to this article can be found online.
