# Supplementary material for: Molecular evolution of intestinal-type early gastric cancer according to Correa cascade
Source: J Biomed Res. 2024 Sep 24;39(3):270–85. doi: 10.7555/JBR.38.20240118 (PMC12239980; doi:10.7555/JBR.38.20240118)
Supplement: Supplementary file 1 — Supplementary data to this article can be found online. [file jbr-39-3-270-Supplementary.pdf]

Available online at www.jbr-pub.org.cn

Open Access at PubMed Central

JBR

Journal of Biomedical Research, 2025 39(3): 1–8

Supplementary Data

Molecular evolution of intestinal-type early gastric cancer according to Correa cascade

Fangyuan Li[1,△], Yaohui Wang[2,△], Xiaochun Ping[3,4,△], Jiani C. Yin[5], Fufeng Wang[5], Xian Zhang[5], Xiang Li[6], Jing Zhai[6], Lizong Shen[3,4,6,✉]

[1]*Digestive Endoscopy Center, Jiangsu Province Hospital of Chinese Medicine, Affiliated Hospital of Nanjing University of Chinese Medicine, Nanjing, Jiangsu 210029, China;*
[2]*Department of Pathology, Jiangsu Province Hospital of Chinese Medicine, Affiliated Hospital of Nanjing University of Chinese Medicine, Nanjing, Jiangsu 210029, China;*
[3]*Department of General Surgery, the First Affiliated Hospital, Nanjing Medical University, Nanjing, Jiangsu 210029, China;*
[4]*Jiangsu Key Lab of Cancer Biomarkers, Prevention and Treatment, Collaborative Innovation Center for Cancer Personalized Medicine, Nanjing Medical University, Nanjing, Jiangsu 211166, China;*
[5]*Geneseeq Research Institute, Nanjing Geneseeq Technology Inc., Nanjing, Jiangsu 210061, China;*
[6]*Department of Surgical Oncology, Jiangsu Province Hospital of Chinese Medicine, Affiliated Hospital of Nanjing University of Chinese Medicine, Nanjing, Jiangsu 210029, China.*

Supplementary materials and methods

The associated scores were determined as follows.

(1) Tumor mutation burden (TMB)

TMB was calculated as the total number of nonsynonymous mutations divided by the length of the genomic target region[1].

(2) Chromosomal instability score (CIS)

CIS was defined as the proportion of DNA segments with a $|\log_2 \text{ratio}| > 0.2$ in all the covered regions of the genome[2].

(3) Loss of heterozygosity (LOH)

Allele-specific copy number analysis was performed using FACETS software (Version 3.1.0, Google Inc., USA). If a segment satisfied total copy number (TCN) > 0 and lesser copy number (LCN) = 0, the segment was considered to have LOH. The LOH score was defined as the proportion of total LOH segments in all the covered regions of the genome[3].

(4) Intratumor heterogeneity (ITH)

Tumor purity was estimated using ABSOLUTE (Version 1.0.6, Broad Institute of MIT and Harvard, USA). The cancer cell fraction (CCF) of mutations was estimated using Pyclone (Version 0.13.1, BC Cancer Research Center, Canada) and FACETS. Mutations with CCF > 0.6 were considered as clonal events and otherwise subclonal. The ITH of a tumor sample was computed as previously described, where the cluster with the largest CCF was considered as the major clone, C_{main}. In cases where only one mutation was detected in this cluster, it was combined with the cluster containing mutations with the second largest CCF. All other clusters were pooled as the subclones C_{sub}. ITH was then calculated as the proportion of subclonal mutations using the following formula[4]:

$$ITH = \frac{N(C_{sub})}{N(C_{main}) + N(C_{sub})}. \quad (1)$$

△These authors contributed equally to this work.

✉Corresponding author: Lizong Shen, Department of General Surgery, the First Affiliated Hospital, Nanjing Medical University, 300 Guangzhou Road, Nanjing, Jiangsu 210029, China; Jiangsu Key Lab of Cancer Biomarkers, Prevention and Treatment, Collaborative Innovation Center for Cancer Personalized Medicine, Nanjing Medical University, 101 Longmian Avenue, Nanjing, Jiangsu 211166, China; Department of Surgical Oncology, Jiangsu Province Hospital of Chinese Medicine, Affiliated Hospital of Nanjing University of Chinese Medicine, 155 Hanzhong Road, Nanjing, Jiangsu 210029, China. E-mails: shenlz@njmu.edu.cn and shenlz@njucm.edu.cn.

Received: 20 April 2024; Revised: 06 September 2024; Accepted: 14 September 2024; Published online: 24 September 2024

CLC number: R735.2, Document code: A

The authors reported no conflict of interests.

This is an open access article under the Creative Commons Attribution (CC BY 4.0) license, which permits others to distribute, remix, adapt and build upon this work, for commercial use, provided the original work is properly cited.

© 2025 by Journal of Biomedical Research.

https://doi.org/10.7555/JBR.38.20240118

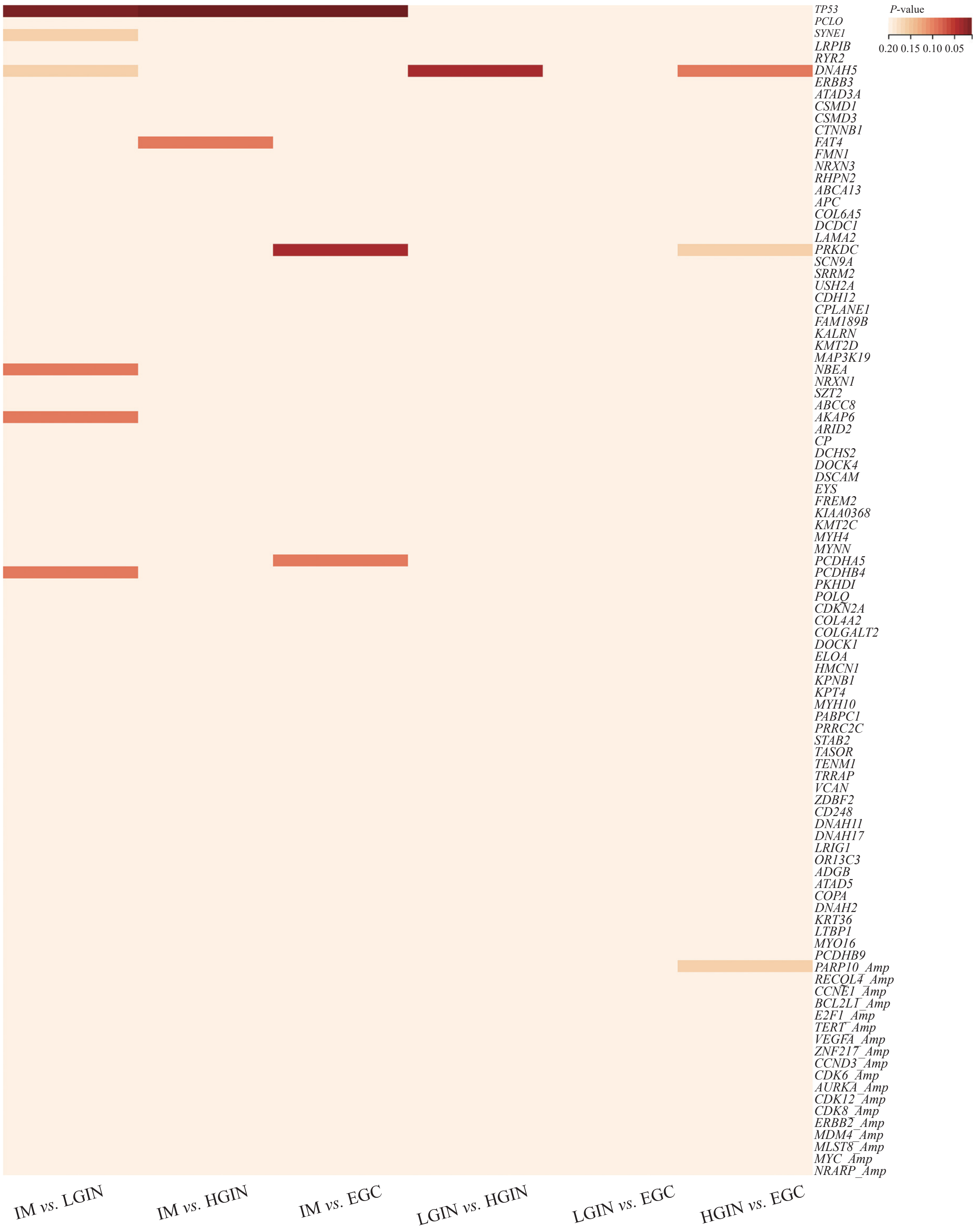

Supplementary Fig. 1 **Comparisons of gene mutation frequencies across the four Correa stages.** IM stage showed dissimilarity to the following LGIN, HGIN, and EGC stages at the gene mutation level. *TP53* was the gene with the most significant difference between IM and other groups. Abbreviations: EGC, early gastric cancer; IM, intestinal metaplasia; LGIN, low-grade intraepithelial neoplasia; HGIN, high-grade intraepithelial neoplasia.

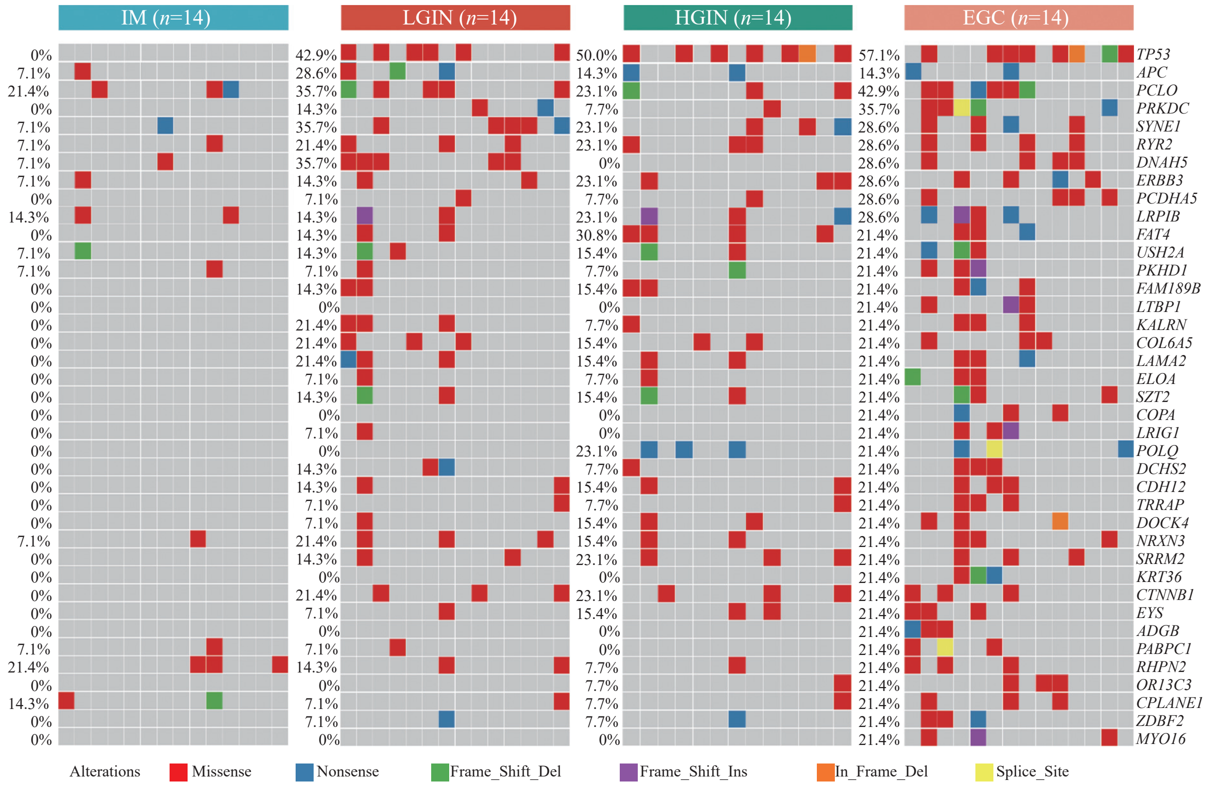

Supplementary Fig. 2 **Genetic landscape of gastric carcinogenesis according to the recurrently mutated genes in EGC.** The patient samples at various stages of gastric cancer development were profiled. Recurrently mutated genes in EGC (occurring in at least three of fourteen patients) were indicated in the analysis. Abbreviations: EGC, early gastric cancer; IM, intestinal metaplasia; LGIN, low-grade intraepithelial neoplasia; HGIN, high-grade intraepithelial neoplasia.

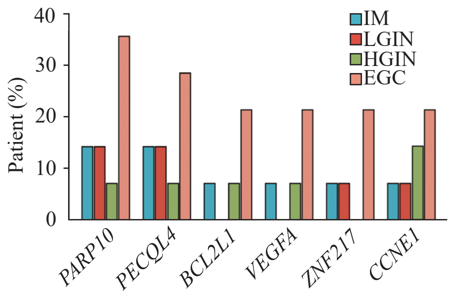

Supplementary Fig. 3 **Disease-wise comparisons of the most frequently identified CNV events in EGC.** The intergroup CNV levels at the single-gene level were analyzed. EGC harbored a trend of increased CNV events, but no difference was found between the four Correa stages. Abbreviations: EGC, early gastric cancer; IM, intestinal metaplasia; LGIN, low-grade intraepithelial neoplasia; HGIN, high-grade intraepithelial neoplasia; CNV, copy number variation.

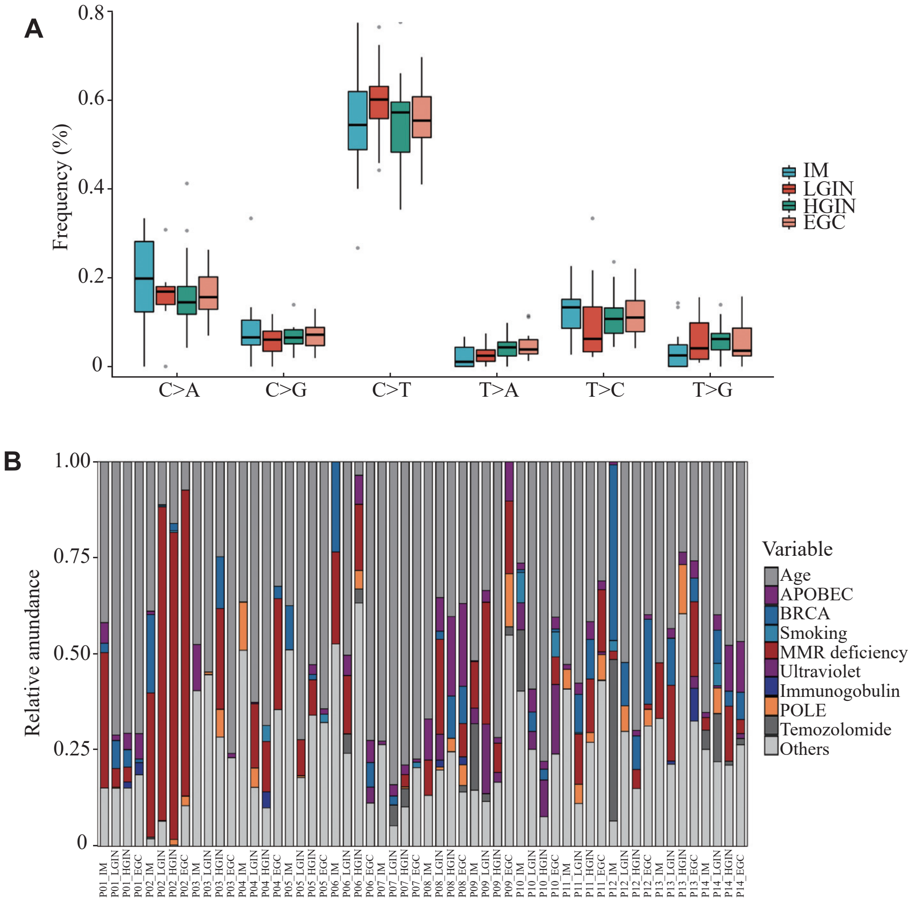

Supplementary Fig. 4 **Mutational signatures associated with early gastric cancer development.** A: Single-nucleotide substitution signatures comparing IM, LGIN, HGIN, and EGC. B: Comparisons of the 30 signatures of mutational processes from the Catalog of Somatic Mutation in Cancer (COSMIC) database. Abbreviations: EGC, early gastric cancer; IM, intestinal metaplasia; LGIN, low-grade intraepithelial neoplasia; HGIN, high-grade intraepithelial neoplasia; MMR, mismatch repair; APOBEC, apolipoprotein B mRNA editing catalytic polypeptide-like; BRCA, breast cancer; POLE, DNA polymerase epsilon.

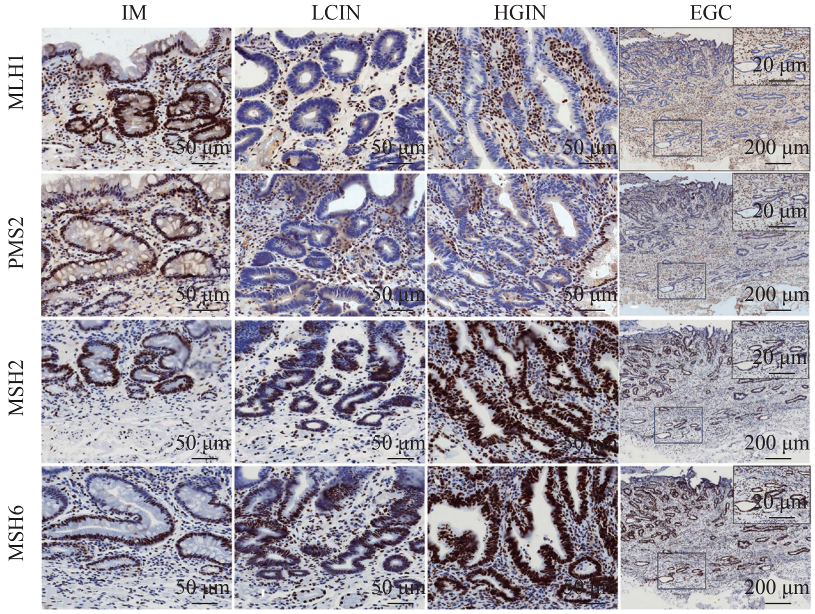

Supplementary Fig. 5 **Immunohistochemistry assay assay of MMR proteins in patient #02.** MMR proteins MLH1, PMS2, MSH2, and MSH6 were retained in the IM area. However, MLH1 and PMS2 were deficient in LGIN, HGIN and EGC lesions, while MSH2 and MSH6 were detected in LGIN, HGIN and EGC lesions. In EGC, the small frames were enlarged in the right upper big frames. Abbreviations: EGC, early gastric cancer; IM, intestinal metaplasia; LGIN, low-grade intraepithelial neoplasia; HGIN, high-grade intraepithelial neoplasia.

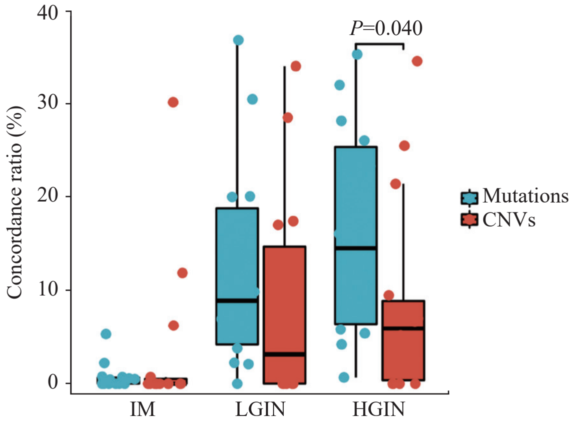

Supplementary Fig. 6 **Combined analysis of overall similarities to EGC at the mutational and CNV level.** IM showed the lowest similarity to EGC at both the mutational and CNV levels. While no significant difference in concordance ratios was observed comparing mutation and CNV assessments in both the IM and LGIN groups, HGIN showed a higher level of mutational similarity as opposed to CNV (P = 0.040). Abbreviations: IM, intestinal metaplasia; LGIN, low-grade intraepithelial neoplasia; HGIN, high-grade intraepithelial neoplasia; CNV, copy number variation.

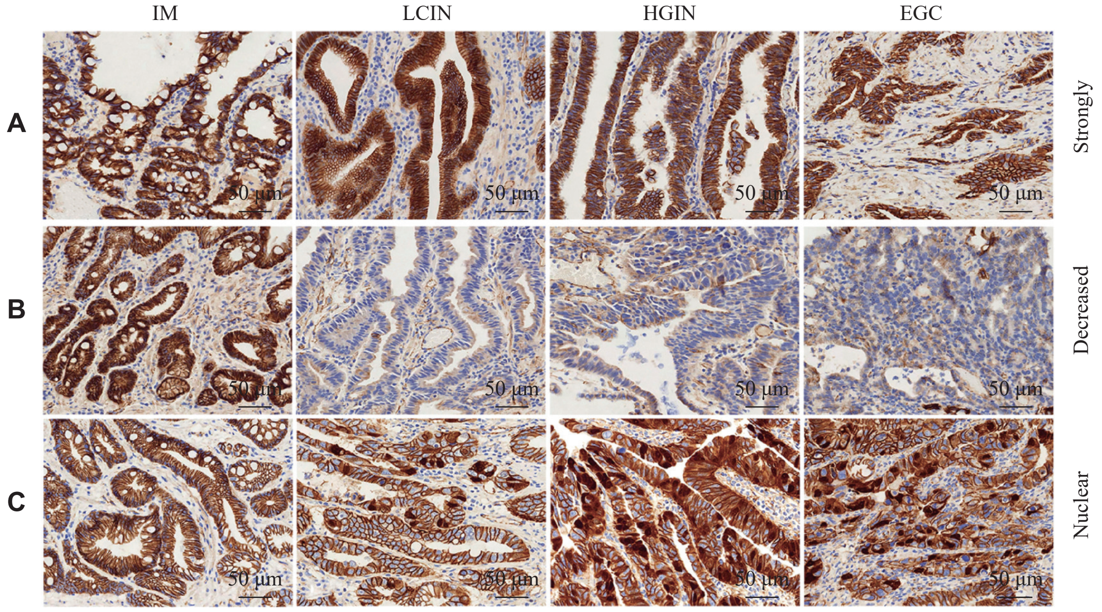

Supplementary Fig. 7 **Immunohistochemistry assay of β-catenin in EGC.** Three types of β-catenin in EGC. A: β-Catenin was strongly expressed in the membrane/cytoplasm among IM, LGIN, HGIN, and EGC. B: Expression of β-catenin was decreased in LGIN, HGIN, and EGC areas, compared with IM. C: β-Catenin was partially expressed in the nucleus of LGIN, HGIN, and EGC areas. Abbreviations: EGC, early gastric cancer; IM, intestinal metaplasia; LGIN, low-grade intraepithelial neoplasia; HGIN, high-grade intraepithelial neoplasia; CNV, copy number variation.

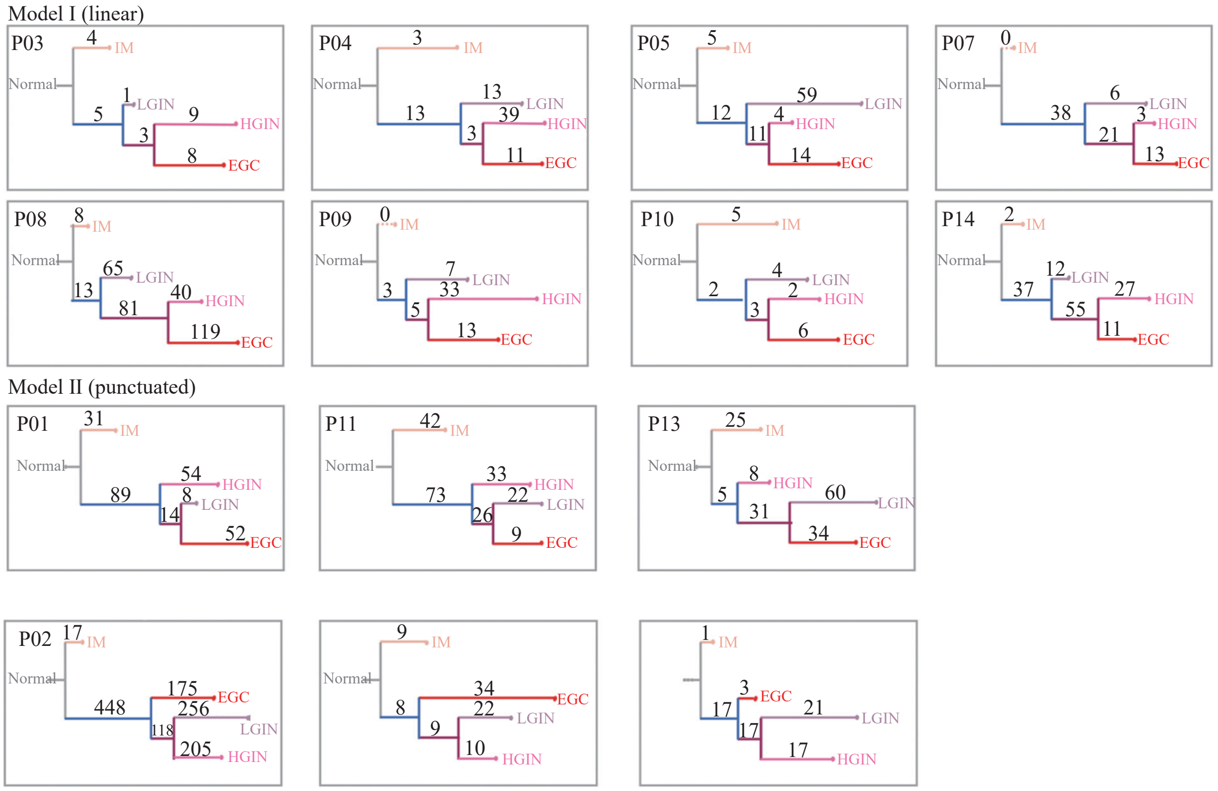

Supplementary Fig. 8 **The case-by-case analyses of evolutionary trajectory.** Treeomics results illustrating the clonal relations between lesions of EGC across the three evolutionary models. The simulated tumor phylogeny for each case is displayed. Numbers denote independently acquired variants in different lesions. Abbreviations: EGC, early gastric cancer; IM, intestinal metaplasia; LGIN, low-grade intraepithelial neoplasia; HGIN, high-grade intraepithelial neoplasia.

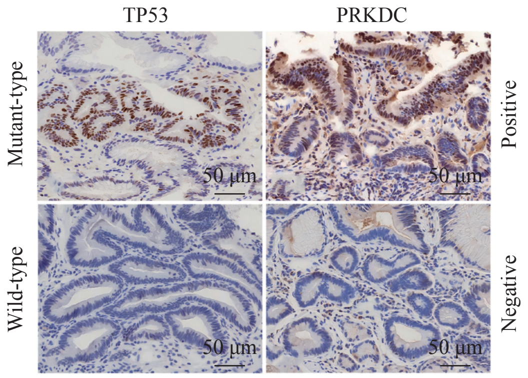

Supplementary Fig. 9 **Immunohistochemistry assays of TP53 and PRKDC in low-grade intraepithelial neoplasia samples.** TP53 expression (mainly in nucleus) suggested mutanted-type, and PRKDC was mainly expressed in the nucleus and cytoplasm.

Supplementary Table 1 **The association of genetic mutations with the Correa stage in each model of evolution**

Models	IM *vs.* LGIN	IM *vs.* HGIN	IM *vs.* EGC	LGIN *vs.* HGIN	LGIN *vs.* EGC	HGIN *vs.* EGC
Model Ⅰ (linear)	0% *vs.* 50%	0% *vs.* 50%	0% *vs.* 50%	50% *vs.* 50%	50% *vs.* 50%	50% *vs.* 50%
	0.080	0.080	0.080	1.000	1.000	1.000
Model Ⅱ (punctuated)			0% *vs.* 100%			
	1.000	0.400	0.100	1.000	0.400	1.000
Model Ⅲ (independent)						
			Inter-group $P > 0.100$			

Analysis of inter-group genetic differences in each evolutionary model was performed. **P* > 0.100 by Wilcoxon rank-sum tests implies there were no differential inter-group genes.
Abbreviations: EGC, early gastric cancer; IM, intestinal metaplasia; LGIN, low-grade intraepithelial neoplasia; HGIN, high-grade intraepithelial neoplasia.

Supplementary Table 2 **The association of *H. pylori* status and the molecular evolution model**

	Model Ⅰ (linear)	Model Ⅱ (punctuated)	Model Ⅲ (independent)	*P*-value
H. pylori-positive	4	0	2	0.370
H. pylori-negative	4	3	1	

Supplementary Table 3 **Clinicopathologic characteristics of the enrolled patients in the validation group**

Subgroups	Patient	Sex	Age (first diagnosis of LGIN, years)	Pathology of ESD specimen	Duration of follow-up (m)	Lesion location	Lesion size (mm)	*H. pylori* infection
**#1**	No.01	M	62	EGC	63	L	11	Negative
**#1**	No.02	F	61	HGIN	46	L	7	Negative
**#1**	No.03	F	72	EGC	48	M	45	Negative
**#1**	No.04	F	54	EGC	34	L	12	Positive
**#1**	No.05	M	69	HGIN	37	U	16	Positive
**#1**	No.06	F	73	HGIN	20	M	11	Negative
**#1**	No.07	M	61	HGIN	13	M	22	Negative
**#1**	No.08	M	68	HGIN	12	L	13	Negative
**#1**	No.09	M	61	HGIN	17	U	26	Negative
**#1**	No.10	M	61	HGIN	52	U	4	Positive
**#1**	No.11	M	64	EGC	77	U	15	Positive
**#1**	No.12	M	72	HGIN	34	L	8	Negative
**#1**	No.13	M	53	HGIN	17	M	3	Negative
**#1**	No.14	M	56	HGIN	19	M	20	Negative
**#2**	No.01	M	78	LGIN	30	L	20	Negative
**#2**	No.02	F	66	LGIN	12	M	9	Positive
**#2**	No.03	M	66	LGIN	14	L	45	Negative
**#2**	No.04	M	63	LGIN	12	M	8	Negative
**#2**	No.05	M	64	LGIN	12	L	8	Positive
**#2**	No.06	F	43	LGIN	49	M	14	Positive
**#2**	No.07	M	62	LGIN	20	M	11	Negative
**#2**	No.08	F	52	LGIN	42	M	6	Negative
**#2**	No.09	F	50	LGIN	20	L	8	Negative
**#2**	No.10	F	71	LGIN	45	M	7	Negative
**#2**	No.11	M	69	LGIN	16	L	12	Negative
**#2**	No.12	M	62	LGIN	12	L	8	Negative
**#2**	No.13	F	62	LGIN	56	L	1.5	Negative
**#2**	No.14	F	66	LGIN	12	L	6	Positive
**#2**	No.15	M	60	LGIN	24	L	8	Negative
**#2**	No.16	F	73	LGIN	29	U	4	Negative
**#2**	No.17	F	64	LGIN	15	L	8	Negative
**#2**	No.18	F	53	LGIN	28	L	8	Positive
**#2**	No.19	M	78	LGIN	48	L	15	Negative
**#2**	No.20	F	62	LGIN	45	L	10	Negative
**#2**	No.21	F	55	LGIN	12	L	6	Negative
**#2**	No.22	F	55	LGIN	15	L	5	Negative
**#2**	No.23	M	50	LGIN	12	L	5	Negative
**#2**	No.24	M	45	LGIN	18	L	5	Negative
**#2**	No.25	M	56	LGIN	19	L	18	Negative

Subgroup #1: the lesions of LGIN in these 14 patients were aggravated into HGIN or EGC during follow-up.
Subgroup #2: the lesions of LGIN in these 25 patients retained the diagnosis of LGIN during follow-up.
Abbreviations: EGC, early gastric cancer; IM, intestinal metaplasia; LGIN, low-grade intraepithelial neoplasia; HGIN, high-grade intraepithelial neoplasia; F, female; M, male; U, upper 1/3 of the stomach; M, middle 1/3 of the stomach; L, lower 1/3 of the stomach.

References

[1] Sha D, Jin Z, Budczies J, et al. Tumor mutational burden as a predictive biomarker in solid tumors[J]. *Cancer Discov*, 2020, 10(12): 1808–1825.

[2] Zhu C, Zhu L, Gu Y, et al. Genomic profiling reveals the molecular landscape of gastrointestinal tract cancers in Chinese patients[J]. *Front Genet*, 2021, 12: 608742.

[3] Shen R, Seshan VE. FACETS: allele-specific copy number and clonal heterogeneity analysis tool for high-throughput DNA sequencing[J]. *Nucleic Acids Res*, 2016, 44(16): e131.

[4] Fang W, Jin H, Zhou H, et al. Intratumoral heterogeneity as a predictive biomarker in anti-PD-(L)1 therapies for non-small cell lung cancer[J]. *Mol Cancer*, 2021, 20(1): 37.
